# Loss of epigenetic adaptation to a high-fat diet in alpha-synuclein transgenic mice

**DOI:** 10.3389/fncel.2026.1901103

**Published:** 2026-08-05

**Authors:** Vivien Hoof, Samantha L. Schaffner, Kristy Dever, Julie MacIsaac, Carola Rotermund, Michael S. Kobor, Philipp J. Kahle, Thomas Hentrich, Julia M. Schulze-Hentrich

**Affiliations:** 1 Department of Genetic/Epigenetics, Saarland University, Saarbrücken, Germany; 2 Edwin S. H. Leong Centre for Healthy Aging, Faculty of Medicine, University of British Columbia, Vancouver, BC, Canada; 3 Pacific Parkinson’s Research Centre, Djavad Mowafaghian Centre for Brain Health, University of British Columbia, Vancouver, BC, Canada; 4 Division of Neurology, Department of Medicine, University of British Columbia, Vancouver, BC, Canada; 5 Department of Medical Genetics, University of British Columbia, Vancouver, BC, Canada; 6 Centre for Molecular Medicine and Therapeutics, BC Children’s Hospital, Vancouver, BC, Canada; 7 Laboratory of Functional Neurogenetics, Department of Neurodegeneration, Hertie Institute for Clinical Brain Research, University of Tübingen, Tübingen, Germany; 8 German Center for Neurodegenerative Diseases (DZNE), Tübingen, Germany

**Keywords:** alpha-synuclein, DNA hydroxymethylation, DNA methylation, epigenetics, high-fat diet, Parkinson’s disease

## Abstract

**Introduction:**

In humans, a high-fat diet and obesity are associated with a higher risk and accelerated progression of Parkinson’s disease (PD). Similarly, in animal models a high-fat diet exacerbates PD-related phenotypes, including dopaminergic neurodegeneration, and alpha-synuclein aggregation. We previously demonstrated that transgenic mice overexpressing human, mutated A30P alpha-synuclein failed to transcriptionally adapt to metabolic stress which could be a potential explanation for the high-fat diet-dependent aggravation of PD pathology. However, the underlying epigenetic mechanisms that might regulate this impaired response remained unknown.

**Methods:**

Here, we profiled genome-wide DNA methylation and hydroxymethylation in brainstem and hippocampus of wild type and transgenic mice exposed to a long-term standard or high-fat diet.

**Results:**

Wild type mice displayed pronounced diet-dependent adaptations that were largely missing in transgenic mice. In the brainstem, a high-fat diet increased the epigenetic age and induced a loss of DNA methylation of neuronal genes involved in protein degradation and mitochondrial metabolism—changes that were largely driven by DNA hydroxymethylation and absent in transgenic mice. Integration of methylation and gene expression data further revealed shared, and brain region-specific interaction networks implicated in metabolism, proteostatis, and neuronal pathways showing molecular adaptation specifically in wild type mice upon high-fat diet.

**Discussion:**

Together, these findings point to failure of high-fat diet-induced epigenetic adaptability under alpha-synuclein overexpression, suggesting that altered DNA methylation and DNA hydroxymethylation might contribute to diet-dependent acceleration of PD pathology.

## 1 Introduction

Parkinson’s disease (PD) is one of the most common neurodegenerative disorders affecting the aging population (Tysnes and Storstein, 2017). Its etiology is multifactorial and based on a complex and still largely enigmatic interplay of genetic predisposition, aging, sex, and environment (Emamzadeh and Surguchov, 2018; Wassouf and Schulze-Hentrich, 2019). The pathological hallmark of PD is the increased accumulation and aggregation of alpha-synuclein protein (aSyn) encoded by the *SNCA* gene (Spillantini et al., 1997; Spillantini and Goedert, 2000; Dauer and Przedborski, 2003). Genetic evidence underscores the fundamental role of aSyn in PD pathogenesis, as point mutations, including A30P, A53T, and E46K, as well as genomic multiplications of *SNCA* are associated with familial cases of PD (Polymeropoulos et al., 1997; Krüger et al., 1998; Singleton et al., 2003; Chartier-Harlin et al., 2004; Zarranz et al., 2004; Greenbaum et al., 2005; Simón-Sánchez et al., 2009; Proukakis et al., 2013) and enhance aSyn fibrilization (Conway et al., 1998; Narhi et al., 1999).

Emerging epidemiological evidence highlights the influence of nutritional factors, including high-fat diets (HFD), in modulating PD pathology (Seidl et al., 2014). Specifically, heightened intake of saturated fat as well as increased total energy consumption are associated with an elevated risk and acceleration of PD progression in humans (Logroscino et al., 1996; Chen et al., 2003; Gao et al., 2007; Qu et al., 2019; Hantikainen et al., 2022). In animal models, a HFD, rich in saturated fat-derived calories, induces insulin resistance, thereby mimicking type 2 diabetes mellitus (Winzell and Ahrén, 2004), which is linked to a more aggressive PD phenotype (Chohan et al., 2021). In addition, prolonged HFD exacerbates PD progression in various PD mouse models: In toxin-induced PD models, a HFD leads to increased neuroinflammation and dopamine depletion as well as an aggravated vascular pathology (Bousquet et al., 2012; Elabi et al., 2021). Studies in genetic mouse models expressing human mutant h[A30P]aSyn (Kahle et al., 2000) demonstrate that HFD accelerates onset of the locomotor phenotype, is accompanied by premature astrogliosis, and earlier aSyn pathology (Rotermund et al., 2014).

Previously, we proposed the failure of metabolic adaptation in several brain regions under the challenge of a HFD as a potential explanation for the accelerated disease progression (Kilzheimer et al., 2023). By examining the brainstem and hippocampal transcriptome of h[A30P]aSyn transgenic mice (TG) and wild type controls (WT) exposed to a life-long HFD, we show a transcriptional adaptation of WT mice under HFD, that was largely missing in TG animals. In particular, TG mice failed to show adaptive changes in oxidative phosphorylation, and mitochondrial function (Kilzheimer et al., 2023). This impairment suggests increased susceptibility to oxidative stress and mitochondrial dysfunction, potentially underlying the aggravated phenotype observed in TG mice under HFD.

The observed gene expression changes point to upstream regulators with DNA methylation (DNAm) emerging as a key candidate mediating environmentally induced transcriptional responses. Consistently, a HFD has been shown to induce DNAm changes across multiple brain regions in WT mice, particularly affecting genes linked to mitochondrial function, neurodegeneration, and metabolic regulation (Vucetic et al., 2012; Yokoyama et al., 2018; Vander Velden and Osborne, 2022). However, these studies often do not distinguish between DNAm and DNA hydroxymethylation (DNAhm), despite the latter being especially abundant and functionally important in the brain, particularly in neurons (Kriaucionis and Heintz, 2009; Globisch et al., 2010; Wen and Tang, 2014). Emerging evidence suggests that also DNAhm is sensitive to dietary influences as chronic consumption of a HFD has been shown to decrease global DNAhm levels in the brain of WT mice, even prior to weight gain, suggesting a role for DNAhm in early epigenetic adaptation of the brain to dietary stress (McFadden et al., 2023).

In this study, we interrogated DNAm and DNAhm in brainstem and hippocampus of WT animals and h[A30P]aSyn TG mice following long-term HFD exposure. Using the Mouse Methylation BeadChip Array, we identify brain region-, genotype-, and diet-specific epigenetic signatures and link them to gene expression changes that might contribute to HFD-driven aggravated PD pathogenesis.

## 2 Materials and methods

### 2.1 Animals and diet

Male TG mice of C57BL/6 background expressing human mutant *h[A30P]SNCA* under control of the CNS neuron-specific *Thy1* promotor (Kahle et al., 2000) were maintained as a homozygous colony. Male WT controls were derived from the same transgenic outcross with C57BL/6 mice and maintained as a parallel colony. For grouping the mice into either a standard or HFD, randomization within blocks representing litters was used to mitigate any litter bias due to genetic effects. Between 5- and 52 weeks of age, homozygous TG and WT mice were kept either on standard chow diet (SD: 3.8% total fat, 3.1 kcal/g, ssniff R/M H Extrudat; ssniff Spezialitäten GmbH, Soest, Germany) or HFD (22.8% total fat, 4.6 kcal/g, TD.06415 Adjusted Calories Diet 45/Fat; Teklad Custom Research Diets, Harlan Laboratories, Boxmeer, The Netherlands). Groups of three to four mice were housed in standard cages (365 × 207 × 140 mm, Typ II long) with normal light/dark cycle (12 h light/12 h dark) and free access to food and water. To minimize unwanted gene expression and DNAm changes during sample collection, WT and TG mice were sacrificed with cervical dislocation followed by head decapitation within 2 min from disturbing the home cage. Brain regions were immediately dissected on ice and snap frozen in liquid nitrogen.

### 2.2 RNA and DNA isolation and mouse methylation bead Chip Array

Total RNA and DNA from brainstem and hippocampus (*n* = 6 animals for each of the four experimental groups per brain region) were simultaneously extracted using the AllPrep DNA/RNA Mini Kit (Qiagen) using the manufacturer’s protocol. RNA library preparation and sequencing was performed as described previously (Kilzheimer et al., 2023). Isolated DNA of three to four animals per experimental group per brain region were bisulfite converted and oxidative bisulfite converted using the NuGEN TrueMethyl oxBS Module (NuGEN Technologies, San Carlos, CA, USA). 200 ng of bisulfite- or oxidative bisulfite-converted DNA per sample were run on Infinium Mouse Methylation BeadChip arrays (Illumina, San Diego, CA, USA) according to the manufacturer’s instructions. Beta values were generated from raw intensity signals for 287,050 CpG sites (~4,000 CpH) using GenomeStudio software (Illumina).

### 2.3 Preprocessing and quality control

Preprocessing of raw methylation data was performed using Minfi (v 1.50.0) (Aryee et al., 2014). Data were normalized using SWAN normalization (Maksimovic et al., 2012). CpG probes with detection *p*-value below 0.05, a standard deviation value below 0.005 across samples, and CpH sites and SNP probes were excluded, leaving 280,759 CpG probes for subsequent analysis. Quality control was performed using RnBeads (v2.22.0) (Müller et al., 2019), and the BeadArray Control Reporter from Illumina. To further confirm absence of outlier samples, principal component analysis (PCA) was performed on normalized methylation data. Pearson correlation coefficients were calculated to evaluate consistency between technical replicates. For downstream analysis, mean methylation values across replicates were used (correlation coefficient between replicates ≥ 0.992). Epigenetic age was calculated using a linear model based on the *Perez-Correa* clock, which is based on 105 CpG probes on the Mouse Methylation Array (Perez-Correa et al., 2022).

### 2.4 Differential analysis and pathway enrichment

Limma (v3.60.6) (Ritchie et al., 2015) was used for differential methylation analysis. DNAm and DNAhm were modelled in a 2 × 2 factorial design as a function of genotype, diet, and their interaction. Significance thresholds for differentially methylated CpGs were set to a BH-adjusted *p*-value ≤ 0.05. Combined rank analysis was based on BH-adjusted p-value and |∆beta|. Both were performed on bisulfite converted samples (Bis) representing DNAm and DNAhm as well as on DNAm and DNAhm values individually. DNAm values were represented by OxBis beta-values. DNAhm was determined by subtracting OxBis beta-value and Bis beta-value at each probe for each sample. The 95% quantile of negative DNAhm values generated after subtraction were applied as detectability threshold (Lunnon et al., 2016). To annotate CpG probes to genomic regions, the R packages IlluminaMouseMethylationanno.12.v1.mm10 (v0.0.2) and annotatr (v1.30.0) (Cavalcante and Sartor, 2017) with the mm10 genome assembly as reference were used. For pathway enrichment, rGREAT (v2.6.0) (Gu and Hübschmann, 2023) was used to identify Gene Ontology terms (Molecular Function, Cellular Component, and Biological Processes) overrepresented among genomic regions of differential CpGs. Annotated genes were tested for enrichment among murine cell type–specific marker genes (McKenzie et al., 2018) using one-sided Fisher’s exact tests.

### 2.5 Analysis of transcriptome data

Transcriptome data from the same animals were processed using the Nf-core RNA-seq pipeline (v3.14) (Ewels et al., 2020). Read quality was assessed using FastQC (v0.12.0) (Andrews, 2010). Reads were aligned against a custom-build reference genome of the Ensembl *Mus musculus* genome (v104) including the human *SNCA* transgene using STAR (Dobin et al., 2013). Normalized read counts were obtained with Rsubread (v2.12.3) (Liao et al., 2019). DESeq2 (v1.44.0) (Love et al., 2014) was used for differential gene expression analysis. Transcripts with less than 20 median reads across samples were excluded from subsequent differential analysis resulting in 15,534 genes for brainstem and 15,465 genes for hippocampus. Gene expression was modelled in a 2 × 2 factorial design as a function of genotype, diet, and their interaction. Significance thresholds for differentially expressed genes were set to BH-adjusted *p*-value ≤ 0.05 and |log_2_FC| ≥ 0.3. Surrogate variable analysis (v3.52.0) (Leek et al., 2012) was applied to remove unwanted variation in the data. MuSiC (v1.0.0) (Wang et al., 2019) was used to deconvolute the transcriptome data using cell-type-specific single-cell RNA-seq reference data (Zeisel et al., 2015). nRPKM values (normalized Reads Per Kilobase per Million total reads) were calculated using read counts from DESeq2 to measure relative gene expression changes (Srinivasan et al., 2016).

### 2.6 Integration of DNA methylation and transcriptome data

Transcriptome data and methylation data were integrated with SMITE (v1.32.0) (Wijetunga et al., 2017) using the murine STRING protein interaction network as reference (v2.16.4) (Szklarczyk et al., 2023). In brief, for each gene an integrative significance score was calculated based on adjusted *p*-values and log_2_ fold change for RNA-seq data and adjusted *p*-values and delta beta-values for DNAm data from bisulfite-treated samples. To focus on gene expression and methylation changes reflecting failed adaption in TG mice upon HFD, effects sizes and significance values of genotype-HFD and diet-WT contrast were used for transcriptome and array data, respectively. Genomic regions were split into promotor (± 1,000bp from TSS), and gene body (TSS + 1,000bp to TES) and p-values were weighted based on genomic regions and the dataset (promoter DNAm = 0.4, gene body DNAm = 0.2, gene expression = 0.4). The SMITE parameters were set to bidirectional for both promoter and gene body methylation, excluding bias toward inverse relationships between methylation and expression. Spinglass algorithm was used to identify significant modules within the protein interaction network showing significantly altered DNAm and expression changes. Modules were further visualized with Cytoscape (Shannon et al., 2003) and annotated by performing pathway enrichment for Biological Processes and Molecular Functions from the Gene Ontology database and KEGG pathways using clusterProfiler (v4.17.0) (Yu et al., 2012).

## 3 Results

### 3.1 Region-specific DNA methylation profiles in brainstem and hippocampus

**Figure 1:**
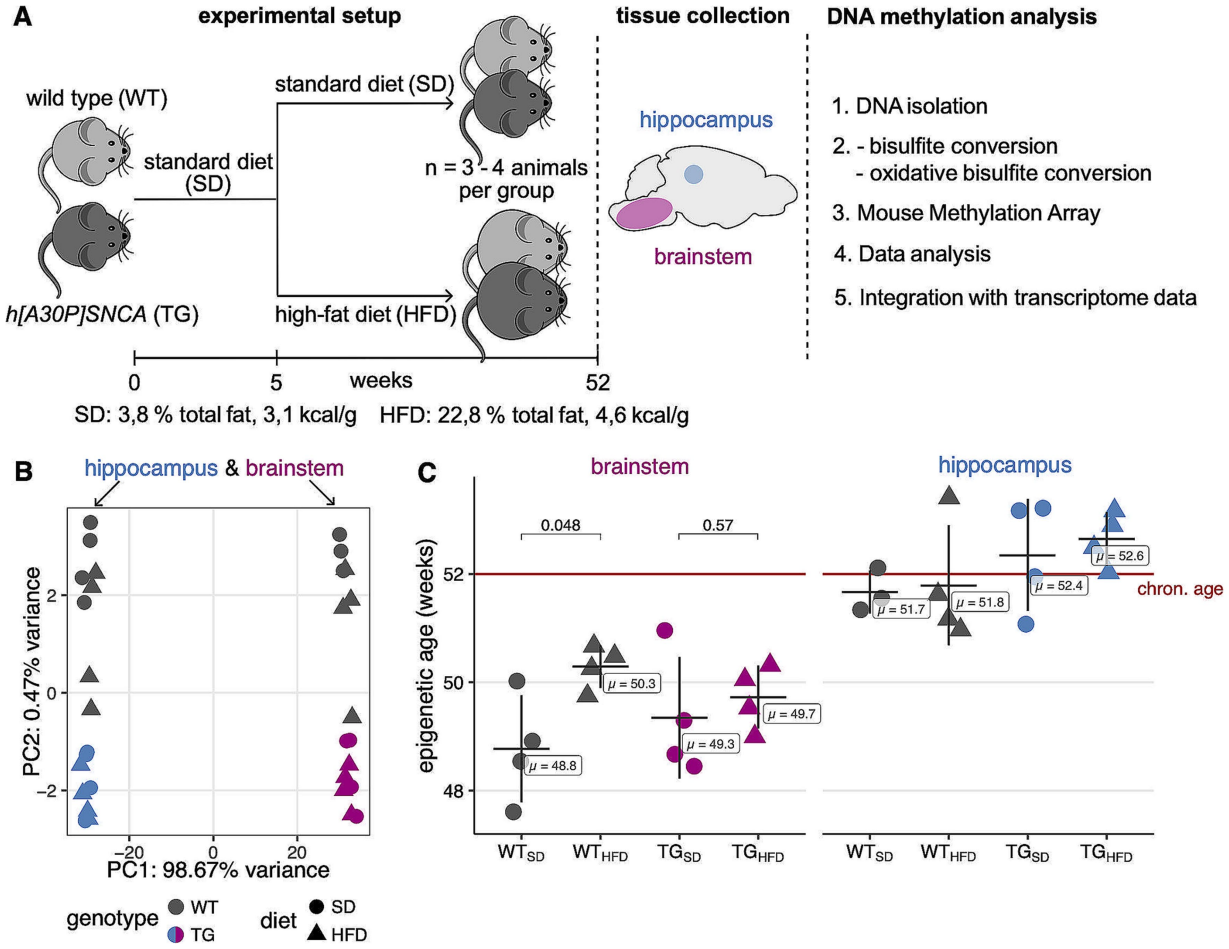
Brain region-specific DNA methylation profiles and distinct epigenetic ages in brainstem and hippocampus. **(A)** Schematic illustration showing the experimental design of WT and TG mice expressing *h[A30P]SNCA* fed a SD or HFD from the age of 5 weeks till 12 months of age. Isolated DNA from brainstem and hippocampus from three to four animals per group was bisulfite or oxidative bisulfite converted and analyzed with the Mouse Methylation Bead Chip Array. **(B)** Principal component analysis of top 1000 most variable CpG sites for all bisulfite converted samples. The percentages along the axes represent variance explained between groups for first and second principal component. **(C)** Epigenetic age calculated per animal using the murine *Perez-Correa* clock based on 105 CpG sites (Perez-Correa et al., 2022), with indicated chronological age. Horizontal and vertical lines represent mean epigenetic age per group and standard error of the mean. Statistical comparisons between groups were performed using unpaired two-tailed T-test.

To study the effects of long-term high energy and fat consumption in a PD mouse model, TG mice expressing human, mutated *h[A30P]SNCA* under the neuron-specific *Thy1* promotor and WT controls were fed a standard chow diet (SD: 3.8% total fat, 3.1 kcal/g) or a HFD (22.8% total fat, 4.6 kcal/g) from 5 weeks onward until the age of 12 months (Figure 1A). As reported in previous studies, mice on HFD weighed about 50 g (48–61 g) in contrast to 35 g (33–41 g) for mice on a SD, without any weight difference between WT and TG animals (Rotermund et al., 2014; Kilzheimer et al., 2023). At the age of 12 months, DNA and RNA was extracted simultaneously from both brainstem and hippocampus— brain regions chosen due to strongest aSyn pathology (Rotermund et al., 2014) and known dietary sensitivity, including HFD-induced impairments in learning and memory (Lizarbe et al., 2019; de Paula et al., 2021). DNA from three to four animals per experimental group was bisulfite (Bis) and oxidative bisulfite (OxBis) converted, enabling the quantification of total methylation (DNAm and DNAhm), DNAm, and DNAhm. Methylation profiling was performed using the Mouse Methylation BeadChip Array, which captures over 285,000 CpG sites across regulatory regions, with raw data being preprocessed using stringent quality control and normalization procedures (Maksimovic et al., 2012; Aryee et al., 2014; Müller et al., 2019) (Supplementary Figure 1 and Methods). Principal component analysis of Bis and OxBis data showed that samples cluster primarily by brain region, followed by genotype, but not by diet (Figure 1B, Supplementary Figures 2, 3). In addition, cell type deconvolution of transcriptome data from the same animals (Kilzheimer et al., 2023) using a published single-cell RNA-seq reference (Zeisel et al., 2015) showed no significant compositional differences across experimental groups within each brain region (Supplementary Figure 4), suggesting that epigenetic and transcriptional differences between groups are unlikely to be driven by changes in cellular composition within each tissue.

Brain regional differences were also reflected in the epigenetic age of the animals. The hippocampal epigenetic age closely matched the chronological of 52 weeks across all experimental groups, with no significant differences between diets or genotypes. In contrast, for brainstem the animals showed a slight overall decrease in epigenetic age compared to the chronological age. Notably, WT animals fed a HFD had a significant diet-induced acceleration of epigenetic aging, while TG mice showed no such increase (Figure 1C), suggesting a genotype-dependent sensitivity to dietary challenges.

### 3.2 HFD-dependent differential methylation in brainstem of WT mice

**Figure 2:**
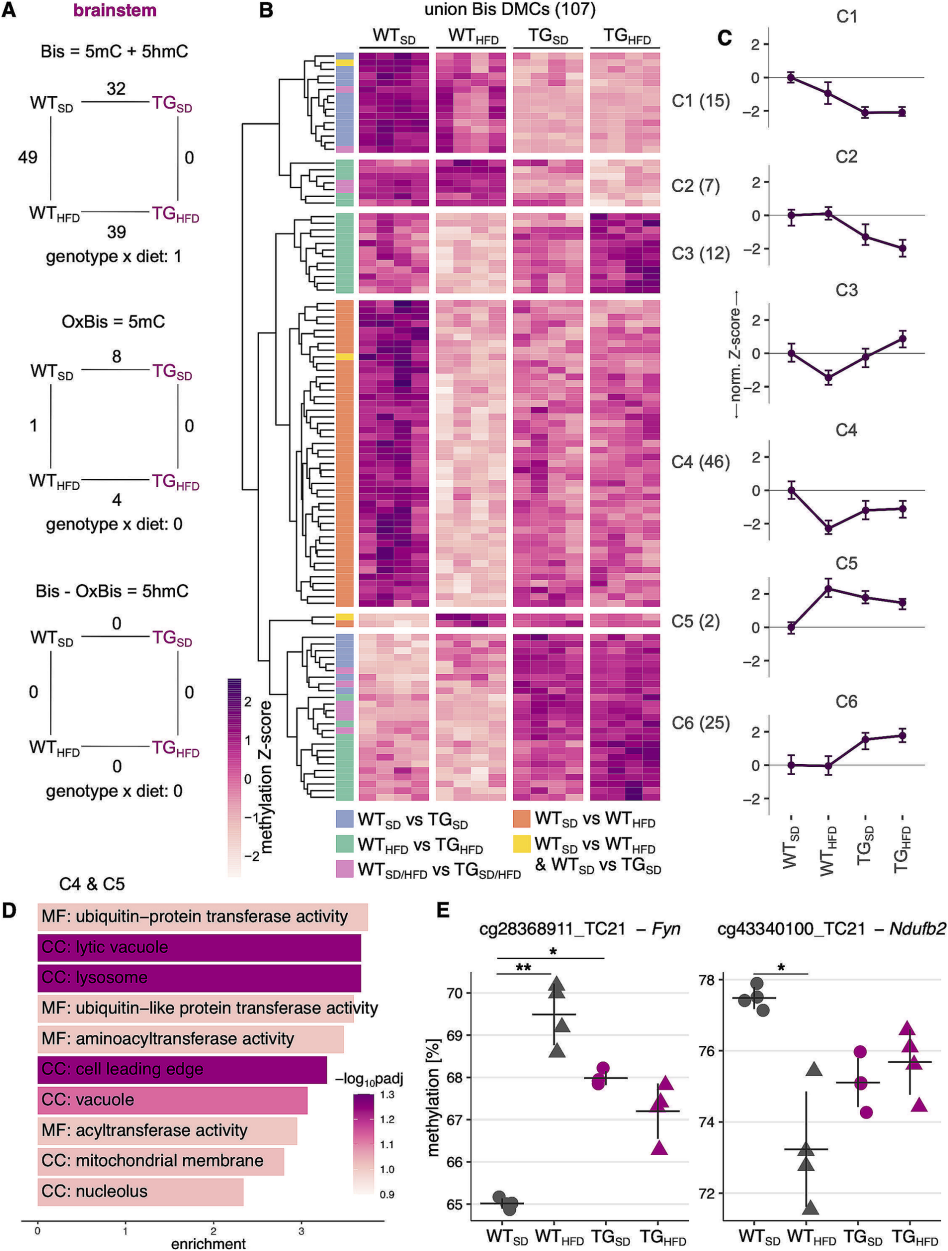
Lack of epigenetic adaptation in TG mice under HFD in brainstem. **(A)** Number of differentially methylated CpG sites (DMCs) between experimental groups in the brainstem, along the genotype (WT and TG) and diet axes (SD and HFD) and their interaction for total methylation, DNAm, and DNAhm only (significance cut-off: P_adj_ ≤ 0.05). **(B)** Heatmap of hierarchically clustered total methylation changes of 107 DMCs identified across all differential comparisons for bisulfite-converted samples across experimental groups as Z-score. CpG annotation shows differential comparison with WT_SD/HFD_ vs. TG_SD/HFD_ representing CpGs differential along the genotype axis for SD and HFD. CpGs marked in yellow are differentially methylated in HFD-fed WT mice and TG mice under SD. **(C)** Average methylation changes and standard deviation of brainstem DMCs as Z-scores per heatmap cluster shown in **(B)** normalized to WT_SD_. **(D)** Ten most significant enriched Gene Ontology terms for DMCs in cluster 4 and 5 in **(B)** with indicated adjusted *p*-value, and enrichment ratio (CC, Cellular compartment; BP, Biological process; MF, Molecular function). **(E)** Total methylation level of cg28368911_TC21 annotated to *Fyn* (p_adj_ = 0.003 between WT_SD_ and WT_HFD_, p_adj_ = 0.04 between WT_SD_ and TG_SD_) and cg43340100_TC21 annotated to *Ndufb2* (p_adj_ = 0.045) in percent across experimental groups including mean and standard error of the mean as horizontal and vertical line. Statistical significance was assessed using limma’s empirical Bayes moderated t-test with Benjamini-Hochberg FDR correction.

To better understand diet- and genotype-driven epigenetic changes in brainstem, differential methylation was determined based on diet, genotype, and their interaction, for total methylation, as well as DNAm and DNAhm separately (Figure 2A). Surprisingly, for bisulfite-treated samples, differentially methylated CpGs (DMCs, p_adj_ ≤ 0.05) were identified only in WT animals fed a HFD, with a total of 49 DMCs (2 hypermethylated, 47 hypomethylated), while no significant DMCs were detected in TG mice under HFD. The lack of DMCs in TG mice under HFD suggests an epigenetic adaptation to dietary changes in WT animals, that TG animals fail to undergo. It should be noted that about two-thirds of the DMCs were annotated to distinctive genes, without any enrichment of promotors and exons compared to the overall array design (Supplementary Table 1; Supplementary Figures 5A–D). Comparing WT and TG animals 32 DMCs were detected under SD and 39 under HFD conditions, of which 10 CpGs overlapped (Supplementary Figure 5E). For DNAm and DNAhm individually, none to very few DMCs were identified (Figure 2A), which might result from higher variability in oxidative bisulfite treated samples (Supplementary Figure 5F).

To contextualize differential methylation upon HFD and transgene overexpression, we visualized methylation profiles across experimental groups. As apparent from the heatmap for the union of DMCs identified in brainstem with respect to total methylation, hierarchically clustering revealed six distinct methylation patterns (Figures 2B,C). The clusters reflected both genotype-driven hypo- or hypermethylation independent of diet (C1, C2, and C6) as well as diet-induced methylation shifts specifically in WT animals (C4, C5). Cluster C3 captured CpGs with inverse methylation pattern between WT and TG mice under HFD, suggesting an opposing effect in TG animals and included CpG cg47193682 annotated to heat shock protein Dnajc13 and CpG cg48113246 annotated to synaptic protein Iqsec2 (Supplementary Table 1).

For the majority of DMCs (C4, C5) only WT mice showed methylation changes in response to HFD, while TG animals lacked a comparable epigenetic adaptation (Figures 2B,C). Functionally the 49 DMCs identified only in WT mice under HFD were enriched for *ubiquitin-protein transferase activity*, *lytic vacuole*, *lysosome* as well as *mitochondrial membrane* (Figure 2D) including cg43340100 annotated to *Ndufb2* a subunit of complex I in the electron transport chain (Loeffen et al., 1998) or cg28368911 as the most significant hypermethylated CpG specifically in HFD-fed WT mice, which is annotated to *Fyn*, encoding for a tyrosine kinase implicated in the regulation of cellular responses to oxidative stress (Gao et al., 2009; Kaspar and Jaiswal, 2011) (Figure 2E). Together these findings suggest that methylation changes related to protein degradation and metabolic pathways largely occur diet-dependent in WT animals only, while TG animals lack a similar epigenetic response to HFD.

### 3.3 Hydoxymethylation-dependent adaptation in HFD-fed WT mice is largely absent in TG animals

**Figure 3:**
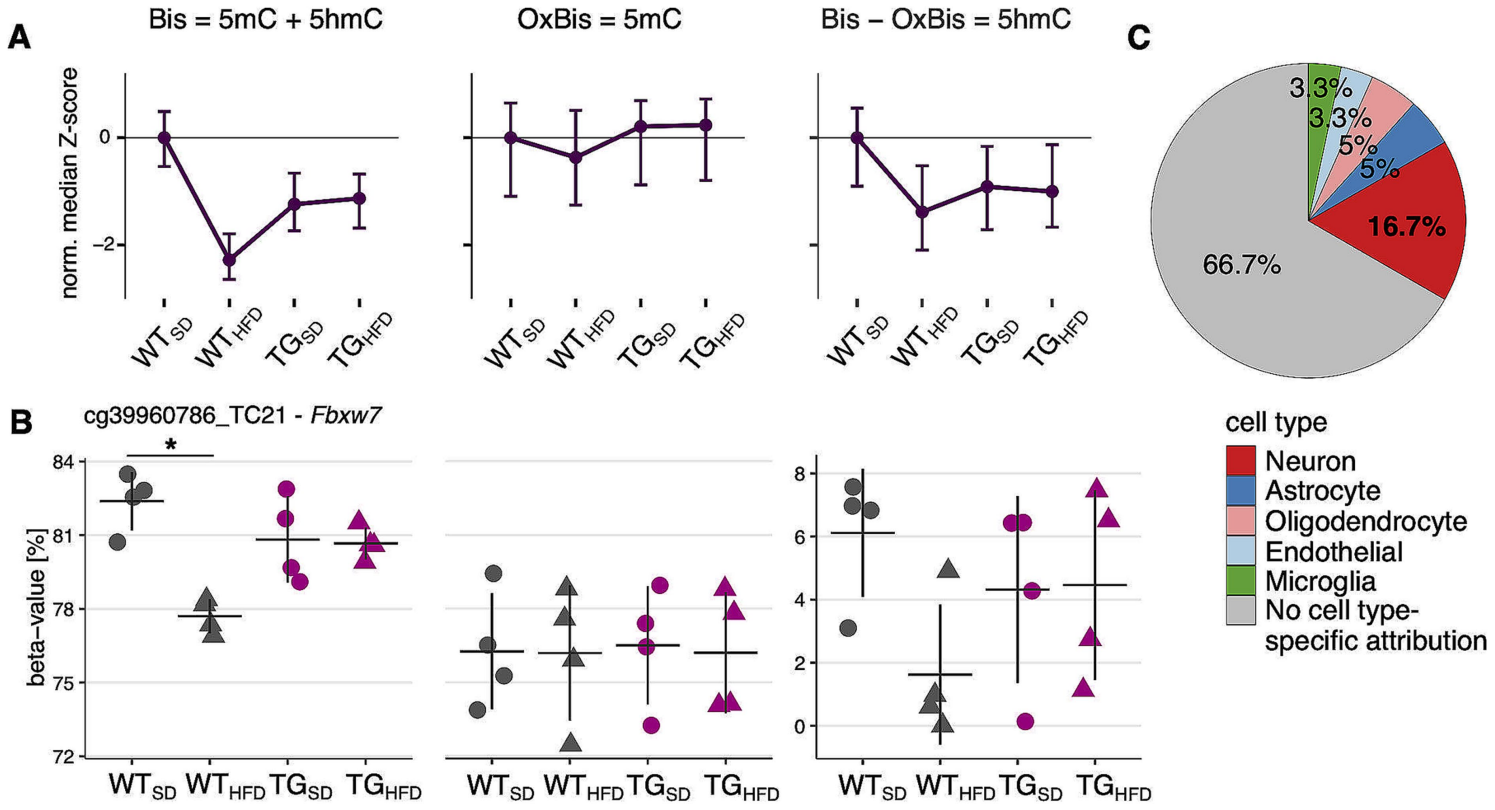
Neuron-specific and hydroxymethylation-dependent adaption to a HFD is absent in TG mice. **(A)** Average methylation changes and standard deviation of brainstem DMCs in diet WT contrast (C1 in Supplementary Figure 6A) plotted as Z-score normalized to WT_SD_ for total methylation, DNAm, and DNAhm only. **(B)** Total methylation, DNAm, and DNAhm levels of cg39960786_TC21 annotated to *Fbxw7* in percent across experimental groups with mean and standard error of the mean as horizontal and vertical line (*p_adj_* = 0.046 for total methylation). Statistical significance was assessed using limma’s empirical Bayes moderated t-test with Benjamini-Hochberg FDR correction. **(C)** Pie chart showing attribution of genes annotated to brainstem DMCs in **(A)** to brain cell types according to cell type-specific reference data from mouse brain (McKenzie et al., 2018).

To better understand the diet-induced epigenetic adaptation of WT mice upon HFD that TG animals fail to undergo, DMCs identified with respect to total methylation were re-examined by separating methylation signals into DNAm and DNAhm (Supplementary Figure 6A). Strikingly, only DNAhm reflected the pattern observed for total methylation (C1 in Supplementary Figure 6A), while no clear pattern emerged for DNAm individually (Figure 3A). Genes annotated to CpGs showing DNAhm-specific changes specifically in WT mice under HFD were enriched for pathways related to protein degradation and mitochondrial membrane (Supplementary Figure 6B), including cg39960786 annotated for ubiquitin ligase *Fbxw7* (Bengoechea-Alonso and Ericsson, 2010) displaying a reduction only of DNAhm in WT mice under HFD conditions, while DNAm remained largely unchanged (Figure 3B). These findings suggest the adaptive epigenetic response to HFD in brainstem in WT animals to be primarily mediated by DNAhm, whereas this regulation appears to be disrupted or lost in TG mice. Importantly, cell type enrichment analysis revealed a significant overrepresentation of neuronal genes underlying DMCs displaying adaptation in WT mice only (Figure 3C, p_adj_ = 0.014), supporting the notion that changes in DNAhm levels are primarily attributed to changes in neuronal cells. In contrast, genotype-driven methylation changes were largely explained by changes in DNAm, rather than DNAhm, and were not enriched for any specific cell type (Supplementary Figure 7).

### 3.4 Distinct epigenetic response to HFD in hippocampus

**Figure 4:**
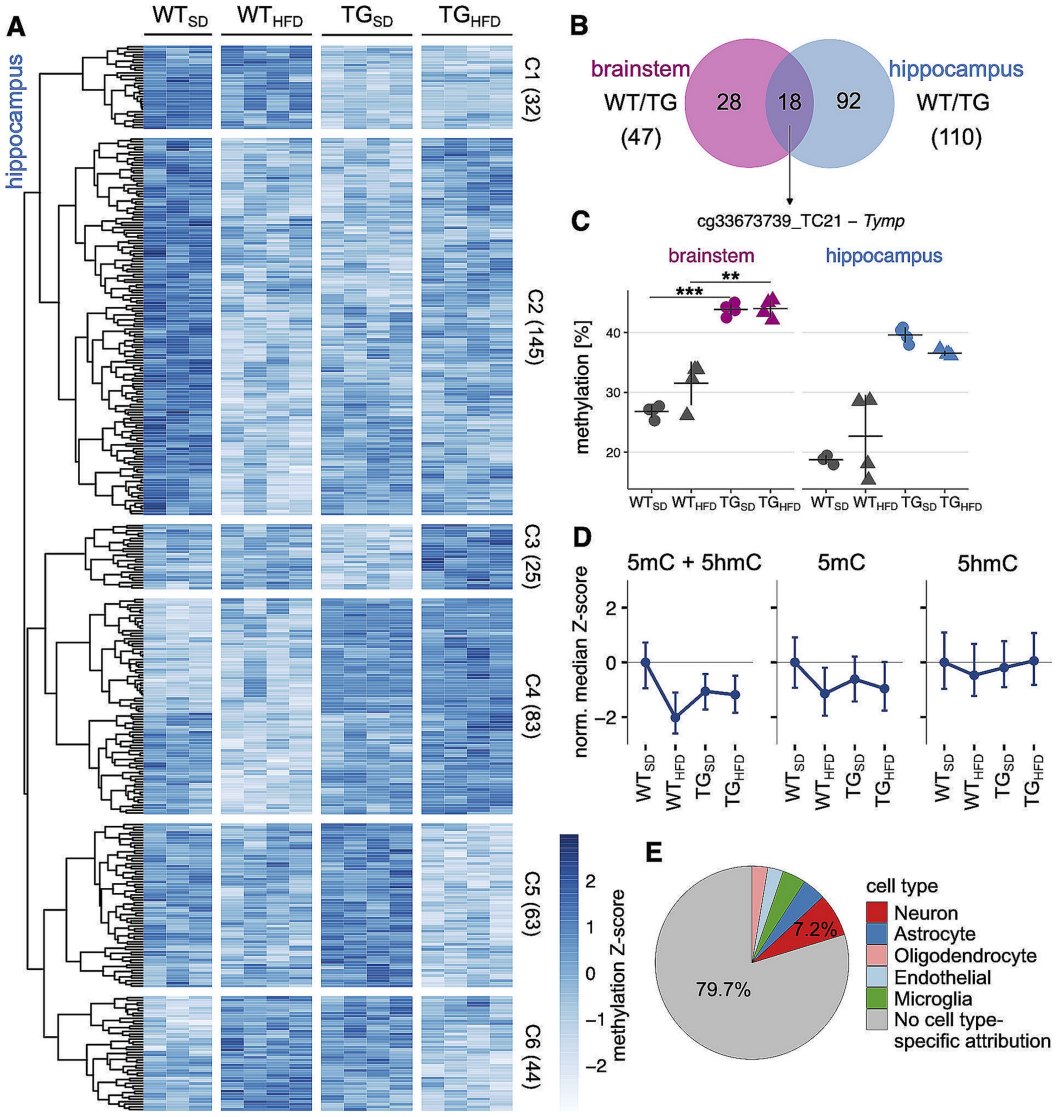
Region-specific and shared methylation changes in hippocampus and brainstem. **(A)** Heatmap of hierarchically clustered total methylation changes of top 100 ranked CpGs across differential all contrasts identified for bisulfite-converted samples across experimental groups in hippocampus. **(B)** Venn diagram comparing 47 differentially methylated CpGs (DMCs) between WT and TG animals for SD and HFD in brainstem and the union of 110 top ranked CpGs in the hippocampus of TG mice. **(C)** Total methylation level of cg33673739_TC21 annotated to *Tymp* across experimental groups and brain regions with mean and standard error of the mean as horizontal and vertical line (*p_adj_* = 0.0002 for genotype SD contrast, *p_adj_* = 0.002 for genotype HFD contrast). Statistical significance was assessed using limma’s empirical Bayes moderated *t*-test with Benjamini-Hochberg FDR correction. **(D)** Average total methylation changes, methylation, and hydroxymethylation changes and standard deviation of top 100 ranked CpGs of diet WT contrast in hippocampus as Z-score normalized to WT_SD_. **(E)** Pie chart showing attribution of genes annotated to top 100 ranked CpGs of diet WT contrast in hippocampus to brain cell types according to cell type-specific reference data from mouse brain (McKenzie et al., 2018).

To identify brain regional specificity of DNA methylation changes upon HFD, hippocampal methylation changes were examined and compared to observations in brainstem. In contrast to brainstem, only none to very few DMCs were identified in hippocampus for total methylation as well as for DNAm and DNAhm individually (Supplementary Figure 8A). Comparing WT and TG mice, only two DMCs were detected under SD and three under HFD for bisulfite-converted samples, with one CpG (cg37319803 - *Serpinb13*) overlapping with DMCs identified in the brainstem (Supplementary Figure 8B). The low number of differential CpGs likely reflects higher variability and the smaller sample size of the hippocampal dataset (Supplementary Figure 8C). Notably, despite the limited number of significant sites, the magnitude of methylation differences was significantly higher in hippocampus compared to brainstem (Supplementary Figure 8D). Therefore, to capture biologically relevant methylation differences beyond the stringent differential threshold, CpGs were ranked by effect size and adjusted significance value (Supplementary Table 2). The union of the top 100 CpGs across all contrasts was visualized in a heatmap (Figure 4A, Supplementary Figure 8E), on which hierarchical clustering revealed six distinct methylation patterns, including genotype-dependent methylation changes (C1 and C4). Top-ranked CpGs from the genotype contrast also overlapped in 18 DMCs identified in brainstem (Figure 4B). Among these shared CpGs, cg33673739, annotated to thymidine phosphorylase *Tymp*, was significantly hypermethylated in TG animals independent of diet and showed similar methylation levels across both brain regions (Figure 4C). In addition, consistent with findings in brainstem, genotype-driven epigenetic changes in hippocampus were primarily attributed to DNAm rather than DNAhm, with no enrichment for a specific cell type (Supplementary Figure 9). This consistency across regions suggests a shared, 5mC-based epigenetic signature associated with transgene overexpression, highlighting the distinct regulatory role of 5mC in mediating genotype-specific methylation changes.

In addition to genotype-driven methylation changes, diet-induced hippocampal methylation changes in WT animals mirrored patterns in brainstem (C2 in Figure 4A). Despite the identical pattern, the underlying CpGs in hippocampus and brainstem were completely disjunct, indicating that HFD-dependent methylation changes are largely region-specific. Furthermore and in contrast to brainstem, hippocampal methylation changes in HFD-fed WT mice were primarily driven by DNAm, not DNAhm, and did not show an enrichment for a particular cell type (Figures 4D,E), highlighting a region- and modification-specific epigenetic response to dietary challenges.

### 3.5 Epigenetic and transcriptional changes with respect to neuronal pathways, protein degradation and metabolism are missing in HFD-fed TG mice

The observed methylation patterns and pathway enrichments in brainstem and hippocampus were largely consistent with our previous transcriptomic findings, which indicates a failure of transcriptional adaptation in TG animals under a HFD (Kilzheimer et al., 2023). To explore potential co-regulation of DNA methylation and gene expression, we determined the overlap between genes annotated to DMCs or top-ranked CpGs and differentially expressed genes (DEGs). Despite repetitive patterns reflecting WT-exclusive adaptation to HFD the overlap was limited, with only one shared gene in brainstem (*Gm15446*) and four in hippocampus (*Igsf21*, *Cntn3*, *Rara*, and *Gm15446*, Supplementary Figure 10A). By relaxing thresholds and including a larger number of top ranked CpGs the overlap extended to 21 DEGs in brainstem, that are linked to metabolic pathways and *aerobic respiration*, and 49 DEGs in the hippocampus enriched for *postsynapse organization*, *postsynapse assembly*, and *regulation of neurogenesis* (Supplementary Figures 10B,C). Among those, several DEGs and CpGs annotated to these genes, including exonic cg28717035 annotated to *Ndufs7*, exonic cg41605845 annotated to *Igsf21*, and intronic cg44295218 annotated to *Apoe*, displayed an inverse relationship between expression and methylation, characterized by reduced methylation and increased expression in WT mice upon HFD, while TG animals lacked both an epigenetic and transcriptional response to HFD (Supplementary Figure 10D).

In addition, we applied SMITE analysis (Significance-based Modules Integrating the Transcriptome and Epigenome), which allows capturing modules of functionally related genes that show coordinated changes in DNAm, gene expression, or both (Wijetunga et al., 2017). For brainstem, a total of 18 significant SMITE modules were identified and 14 for hippocampus, with many modules enriched for metabolism, protein degradation, and neuron-related pathways (Supplementary Table 3). While most modules were region-specific, several shared modules were detected in both brain regions (Supplementary Figure 11A).

**Figure 5:**
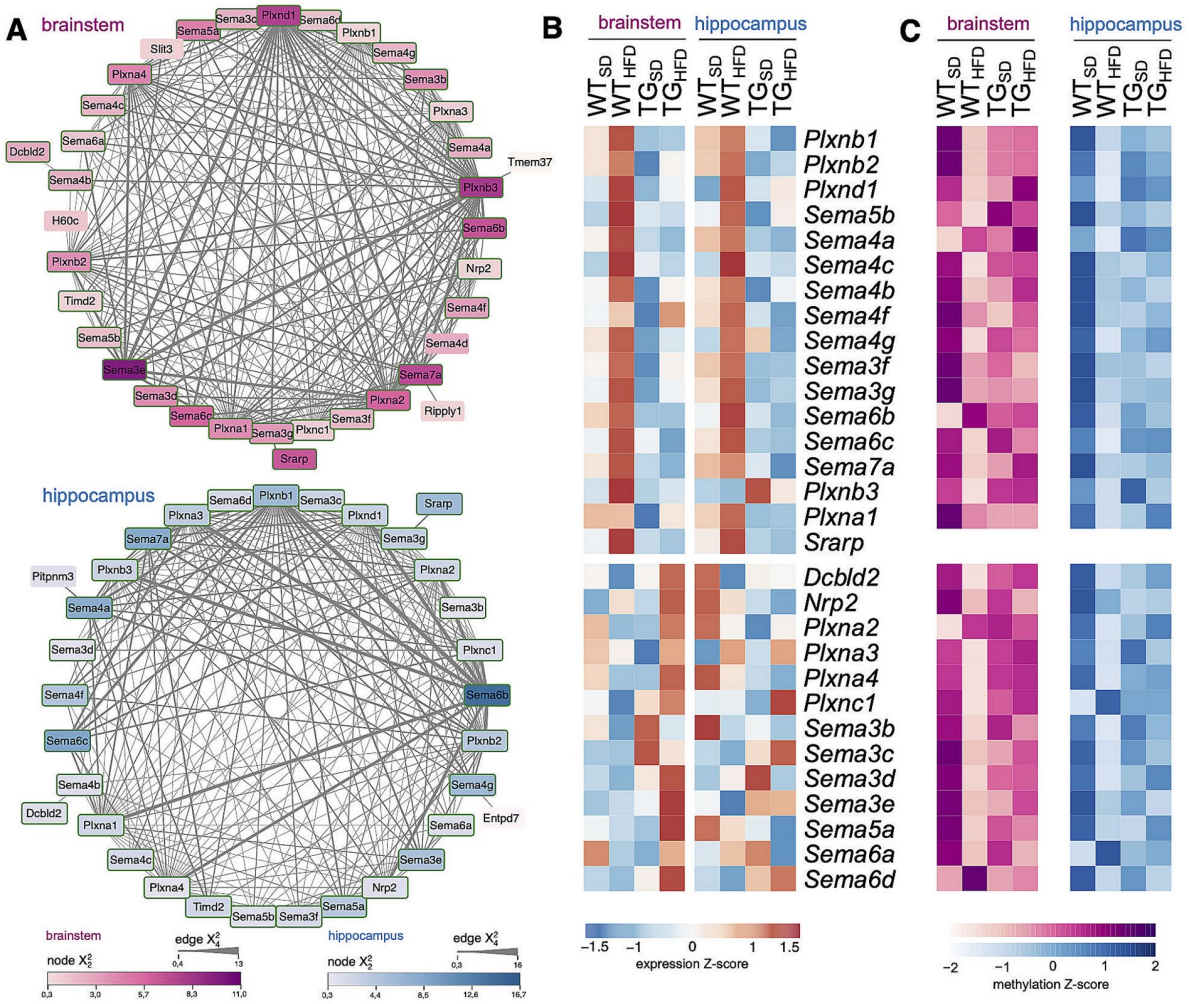
Shared protein interaction network between brainstem and hippocampus largely showing inverse expression and methylation changes. **(A)** Protein interaction network for semaphoring-plexin signaling module identified by SMITE analysis as most significant module in brainstem and second most significant module in hippocampus. Shared network genes are outlined in green. Node color and edge thickness represents chi-square values derived from SMITE representing for the individual gene (node) or the gene connection (edge) how strongly they show evidence for coordinated expression and methylation changes. **(B)** Expression changes per experimental group as Z-scores for 31 shared network genes from module shown in **(A)** for brainstem and hippocampus with indicated gene names. **(C)** Total methylation changes per experimental group as Z-score for top ranked CpG for each shared network gene shown in **(B)** for brainstem and hippocampus.

In the hippocampus, the most significant and region-specific module was enriched for *type I diabetes*, *hormone* and *insulin processing* (Supplementary Figure 11B). Several genes of this module displayed inverse expression and methylation changes, with reduced methylation and increased expression in WT mice under HFD, while TG mice failed to show any transcriptional and epigenetic response (Supplementary Figure 11C). A shared module across regions—the most significant module in brainstem, and the second most significant in hippocampus—was enriched for the *semaphorin-plexin signaling pathway* and *axonogenesis* (Figure 5A; Supplementary Table 3). The majority of genes in this module overlapped between regions (Supplementary Figure 11D), with many of them showing upregulation only in WT mice fed a HFD, reflecting again a failure of transcriptional adaptation in TG animals (Figure 5B). Consistent with these transcriptional differences, the majority of top-ranked CpGs annotated to module genes displayed methylation changes, either inversely or directionally consistent with gene expression patterns (Figure 5C). These coordinated methylation and expression patterns were not due to genomic clustering and cannot be explained by genomic vicinity and potential transgenic integration sites, as genes of the semaphorin-plexin signaling pathway are distributed across the genome (Supplementary Figure 11E).

Together, these results indicate that a HFD induces pathway-specific epigenetic and transcriptional changes in WT mice across brain regions, particularly in neuron-, protein degradation- and metabolism-related processes, whereas TG animals fail to adapt to the dietary challenge on both methylation and gene expression level.

## 4 Discussion

Obesity and HFD, particularly excessive intake of saturated fats, have been implicated in the modulation of PD pathology and are associated with accelerated pathogenesis in PD patients and animal models (Chen et al., 2003; Bousquet et al., 2012; Rotermund et al., 2014; Qu et al., 2019; Elabi et al., 2021; Park et al., 2022). In our previous work, we demonstrate that TG mice overexpressing human mutated aSyn exhibit a HFD-dependent exacerbation of neurodegeneration, potentially based on failed transcriptional adaptation in response to higher energy load (Rotermund et al., 2014; Kilzheimer et al., 2023). In this study, we extended these findings to the epigenetic level and showed a similar absence of adaptive responses in TG mice also for DNAm. While WT mice displayed clear HFD-dependent methylation changes, the methylome of TG animals remained largely unresponsive to high energy intake. Consistently, in brainstem, only WT mice under HFD aged epigenetically faster than under SD. In line, accelerated epigenetic aging has also been observed in previous studies from HFD-fed WT mice (Sandoval-Sierra et al., 2020) and is associated with obesity in humans (Foster et al., 2023; Izquierdo et al., 2025; Jung et al., 2025). Rather than necessarily indicating pathological aging, the accelerated epigenetic age in WT animals upon HFD might reflect an adaptive epigenetic response to higher energy load, suggesting that absence of age acceleration in TG mice might be due to loss of epigenetic adaptability under HFD.

Interestingly, a similar pattern was particularly evident in brainstem, where WT mice under HFD showed differential methylation of genes related to protein degradation and mitochondrial metabolism, whereas TG mice exhibited no comparable response. On transcriptional level, several genes related to proteasomal and lysosomal degradation were similarly upregulated specifically in WT mice under HFD (Kilzheimer et al., 2023). A HFD is known to impair mitochondrial function and increase oxidative stress, leading to enhanced protein damage and misfolding (Pugazhenthi et al., 2016). In line with this, previous studies have reported upregulation and increased activity of the ubiquitin–proteasome system under HFD, which likely represents a compensatory mechanism to maintain proteostasis under conditions of elevated oxidative stress (Ignacio-Souza et al., 2014). Thus, methylation and expression changes of protein degradation-related genes observed here likely reflect an epigenetically mediated adaptive response to counteract increased levels of protein damage imposed by higher energy load. Importantly, TG mice showed neither comparable transcriptional nor epigenetic changes. Since efficient protein degradation is also crucial for clearance of misfolded aSyn, absence of this adaptive epigenetic regulation could promote aSyn accumulation and contribute to accelerated synucleinopathy and the stronger locomotor phenotype described in 17- and 20-month-old A30P mice under HFD (Rotermund et al., 2014).

Interestingly, the majority of differential CpGs displayed modest methylation changes in TG mice under SD, pointing to a genotype-dependent shift in baseline methylation levels that may contribute to reduced adaptability of TG animals to dietary challenges. In addition, a small subset of DMCs showed reverse methylation changes between WT and TG mice under HFD. Although no significant pathway enrichment was detected, likely due to the limited number of CpGs, they were annotated to genes of potential functional relevance, such as the heat shock protein and PD risk gene *Dnajc13* (Vilariño-Güell et al., 2013). Hence, some components of the unfolded protein response may be epigenetically regulated differently under alpha-synuclein overexpression and upon HFD exposure.

Furthermore, among CpGs showing a diet-dependent hypermethylation specifically in WT mice and not in TG animals was cg28368911 annotated to *Fyn*, which was transcriptionally downregulated under HFD. *Fyn* encodes for a tyrosine kinase that negatively regulates the key transcription factor Nrf2, which controls the expression of proteasomal and antioxidant proteins to maintain homeostasis under stress conditions (Kaspar and Jaiswal, 2011; Pajares et al., 2017). In HFD-fed TG mice, the absence of methylation and gene expression changes of *Fyn* might suggest that Nrf2 activity remains suppressed, potentially compromising antioxidant defences.

Similarly, methylation changes in HFD-fed WT mice also affected genes implicated in mitochondrial metabolism including Fbxw7, a key regulator of cellular metabolism interacting for instance with PGC-1α thereby regulating mitochondrial biogenesis and oxidative phosphorylation (Shen et al., 2022). In line, genes involved in mitochondrial respiration, including *Ndufb2* and *Ndufs7*, exhibited reduced methylation and increased expression levels, suggesting an adaptive upregulation of mitochondrial metabolism which might be mediated by reduced methylation levels in response to high energy intake. Although HFD exposure initially introduces mitochondrial damage through enhanced ROS production (Sergi et al., 2019), prolonged HFD promotes mitochondrial biogenesis and increased gene expression of mitochondria- and metabolism-associated genes likely represents a cellular adaptation to cope with higher energy levels (Turner et al., 2007; Hancock et al., 2008; Jain et al., 2014). In the PD mouse model, however, genotype-dependent changes are accompanied by a lack of HFD-induced adaptation on both the epigenetic and transcriptional level, which may underly the aggravated phenotype.

Importantly, epigenetic changes observed in brainstem were largely driven by DNAhm. DNAhm is particularly abundant in neurons (Kriaucionis and Heintz, 2009), consistent with higher TET expression in neuronal compared to glial cells (Antunes et al., 2019). Although glial cell types are heterogenous in their DNAhm content, they generally exhibit lower DNAhm levels than neurons, with microglia having the lowest and astrocytes the highest DNAhm abundance among glial cell types (Tooley et al., 2023). Consistently, genes annotated to CpGs displaying differences in DNAhm levels were significantly enriched for neuronal genes, whereas glia-specific genes contributed to only a small fraction of DNAhm changes. Furthermore, DNAhm is known to regulate neuron-specific gene expression (Marion-Poll et al., 2022), including pathways involved in energy metabolism and protein degradation (Ellison et al., 2017). For the majority of DMCs, TG mice already displayed different baseline DNAhm levels, while HFD-fed WT mice exhibited a strong reduction in DNAhm, in line with previous observations in hypothalamus (McFadden et al., 2023). However, this effect appears to be region-specific, as similar DNAhm-specific changes were not detected in hippocampus despite its higher neuronal content compared to brainstem (Zhang et al., 2023), suggesting region- and modification-specific epigenetic changes under dietary stress. In contrast, genotype-dependent methylation changes were largely driven by DNAm rather than DNAhm. This applied to both regions which also partly overlapped in DMCs, including cg33673739, annotated to thymidine phosphorylase *Tymp*, which has been implicated to mitochondrial dysfunction in a PD mouse model (Ikuno et al., 2021). Future studies will need to address whether these findings extend to brain regions of the dopaminergic system, such as striatum and substantia nigra, since DNAhm levels and *TET2* expression are altered in dopaminergic neurons in PD (Wu et al., 2020).

In contrast to brainstem, only few DMCs were identified in hippocampus in both genotype and diet- comparisons. This may reflect slightly higher variability or a reduced epigenetic sensitivity to HFD of the hippocampus or may also be related to the hippocampus being affected by PD pathology only at later stages (Braak et al., 2003). Nevertheless, analysis of top-ranking CpGs revealed coordinated methylation and expression changes of genes related to neuronal and synaptic functions, including *ApoE* and *Igsf21*. These findings align with previous studies reporting that pathways related to synaptogenesis and nervous system development are affected on epigenetic and transcriptional level in hippocampus and frontal cortex of obese WT mice (Yokoyama et al., 2018; Vander Velden and Osborne, 2022). Additionally, the most significant hippocampal protein interaction module was enriched for insulin processing and included genes such as *Pcsk1n* and *Ptprn* that were hypomethylated and upregulated specifically in HFD-fed WT mice. Although traditionally linked to peripheral metabolism, insulin signaling in hippocampus plays a crucial role in synaptic plasticity and cognition (Stranahan et al., 2008; Fadel and Reagan, 2016) and the epigenetic and transcriptional changes observed herein likely reflect an adaptive response to preserve hippocampal function during metabolic stress. Furthermore, PD is associated with impaired insulin signaling in the brain (Bassil et al., 2022; Foroozanmehr et al., 2025) in line with a slight reduction in transcriptional levels in TG mice compared to WT animals under SD. Adaptive methylation and expression changes observed for WT mice were, however, absent in HFD-fed TG animals and might exacerbate hippocampal vulnerability.

Integration of methylation and transcriptome data further revealed shared gene interaction networks across both brain regions, in which semaphorin-plexin signaling emerged as the most significant pathway showing largely inversely correlated expression and methylation changes. In line, genes of the semaphorin-plexin signaling pathway and CpGs annotated to them largely changed specifically in WT mice upon HFD, whereas no changes were observed for TG animals. Semaphorins and their receptor molecules have been originally identified as axon guidance molecules (Pasterkamp and Giger, 2009), but are also implicated in HFD- and obesity-related metabolic disorders in various peripheral tissues (Lu and Zhu, 2020). In brain, this pathway plays an important role in regulating energy homeostasis as the deletion of semaphorin 3 promotes adiposity and rare variants in semaphoring 3 and their receptors are enriched in severely obese individuals (van der Klaauw et al., 2019). Therefore, the transcriptional activation of semaphorins and plexin molecules, potentially mediated by methylation changes, could be an adaptive response in WT mice to HFD in order to maintain energy homeostasis, which is missing in TG mice. It is important to note, that for the majority of the discussed CpG sites TG animals displayed baseline epigenetic changes for both DNAm and DNAhm. Although these changes did not reach statistical significance in TG mice under standard conditions, they consistently shifted in the same direction as HFD-induced alterations in WT mice. Those changes–observed across both multi-omics layers–may already be sufficient to disrupt signaling pathways and could contribute to the reduced adaptability of the TG animals to dietary challenges.

Taken together, our findings revealed a consistent molecular pattern across methylome and transcriptome and two brain regions, that suggests WT mice to be capable of adapting to HFD while TG animals fail to do so. These adaptations were primarily mediated by DNAhm in neurons of the brainstem and mainly affected proteostatis, mitochondrial, and neuronal functions. Impaired DNAhm-dependent adaptability might therefore underlie the aggravated PD pathology in aSyn TG mice under high energy load.

However, several limitations of the study should be acknowledged. First, the study was performed for a relatively small cohort of three to four male mice, which might limit statistical power and does not capture potential sex-specific responses to HFD or aSyn overexpression. Future studies including larger, sex-balanced cohorts will be important to validate these results. Second, no parallel behavioral or neuropathological analyses were performed for the 12-month-old cohort used in this study. Published pathology and behavioral phenotyping on the effect of a HFD in A30P TG mice has been performed at 17 and 20 months of age (Rotermund et al., 2014), and age-dependent differences in alpha-synuclein pathology may limit direct comparability with findings at 12 months. Third, although our integrative approach highlights impaired DNAhm-dependent adaptability in TG mice, mechanistic work, such as targeted experiments assessing Nrf2 activity and semaphorin-plexin signaling, will be required to validate the pathways linking metabolic stress, epigenetic changes, and aSyn pathology. Fourth, the low direct overlap between DMCs and DEGs limits the ability to infer direct regulatory relationships as transcriptional changes may precede methylation changes, or other epigenetic marks could drive gene expression independently. A notable strength of our study, however, is the distinction between DNAm and DNAhm, which revealed that total methylation changes observed upon HFD in WT mice were in fact driven by DNAhm. This has broader implications for interpreting brain-based methylation studies that rely solely on DNAm, as DNAhm-specific changes may be masked when not profiled separately. Together, these considerations provide important context for future studies aimed at defining how epigenetic adaptation shapes vulnerability to metabolic stress in PD.

## Data Availability

The datasets presented in this study can be found in online repositories. The names of the repository/repositories and accession number(s) can be found at: https://www.ncbi.nlm.nih.gov/geo/, GSE313041.

## Supplementary material

SUPPLEMENTARY FIGURE 1 Density of beta-value distribution for brainstem and hippocampus separated for bisulfite (Bis) and oxidative bisulfite converted (OxBis) samples after normalization.

SUPPLEMENTARY FIGURE 2 Samples separate by brain region and genotype. Principal component analysis of top 1,000 most variable CpG sites for **(A)** bisulfite converted samples (Bis) for brainstem and hippocampus, **(B)** all oxidative bisulfite converted (OxBis) samples, and **(C)** OxBis samples separated for brainstem and hippocampus. The percentages along the axes represent variance explained between groups for first and second principal component.

SUPPLEMENTARY FIGURE 3 Samples largely cluster by genotype. Sample correlation for the top 1,000 most variable CpG sites for bisulfite (Bis) and oxidative bisulfite converted (OxBis) samples for brainstem and hippocampus with indicated Pearson correlation coefficient.

SUPPLEMENTARY FIGURE 4 Estimated relative cell type shares across experimental groups according to deconvolution based on bulk transcriptome data using a scRNAseq reference.

SUPPLEMENTARY FIGURE 5 HFD- and genotype-dependent differential methylation-changes in brainstem. **(A)** Volcano plot for methylation changes in brainstem per contrast. Differential hypomethylated CpGs are colored in blue and differential hypermethylated CpGs in red. **(B–D)** Pie charts showing annotation of CpG sites for genomic regions for **(B)** all CpGs on the mouse bead chip array, **(C)** the union of DMCs identified in the brainstem, and **(D)** DMCs identified for different contrasts. **(E)** Venn diagram comparing 32 differentially methylated CpGs (DMCs) between WT and TG animals for SD and 39 DMCs identified for HFD with overlapping CpGs shown. **(F)** Violin plot showing the variance distribution for the top 1,000 most variable CpG sites for bisulfite converted (Bis) and oxidative bisulfite converted samples (OxBis).

SUPPLEMENTARY FIGURE 6 HFD-induced methylation changes in WT mice are largely driven by changes in DNAhm and are absent in TG animals. **(A)** Heatmap of 49 DMCs identified in the diet WT contrast for bisulfite converted samples in the brainstem across experimental groups for total methylation, DNAm, and DNAhm only. **(B)** Ten most significant enriched Gene Ontology terms for C1 DMCs in **(A)** with indicated adjusted *p*-value, and enrichment ratio (CC, cellular compartment; BP, biological process; MF, molecular function).

SUPPLEMENTARY FIGURE 7 Genotype-dependent methylation changes are largely driven by changes in DNAm. **(A)** Heatmap of 61 DMCs identified in the genotype contrast (SD and HFD) for bisulfite converted samples across experimental groups for total methylation, DNAm, and DNAhm only. **(B)** Pie chart showing attribution of genes annotated to genotype DMCs to brain cell types according to cell type-specific reference data from the mouse brain (McKenzie et al., 2018).

SUPPLEMENTARY FIGURE 8 HFD- and genotype-dependent differential methylation-changes in hippocampus. **(A)** Number of differentially methylated CpG sites (DMCs) between experimental groups in hippocampus, along the genotype (WT and TG) and diet axes (SD and HFD) and their interaction for total methylation, DNAm, and DNAhm only (significance cut-off: p_adj_ ≤ 0.05). **(B)** Venn diagram comparing 61 DMCs between WT and TG animals (SD and HFD) in brainstem and 4 DMCs identified in hippocampus with overlapping CpG, and annotated gene shown. **(C)** Violin plot showing the variance distribution for differentially methylated and top ranked CpGs for brainstem and hippocampus across experimental groups. **(D)** Violin plot showing the distribution of absolute methylation differences of top 100 ranked CpGs per contrast and brain region. Statistical comparisons between groups were performed using unpaired two-tailed T-test. **(E)** Average methylation changes and standard deviation of top 100 ranked CpGs per contrast as Z-scores per heatmap cluster from Figure 4A normalized to WT_SD_.

SUPPLEMENTARY FIGURE 9 Hippocampal methylation changes are largely driven by changes in DNAm. Heatmap of 392 CpGs identified as the union of top 100 ranked CpGs across all contrasts for bisulfite converted samples plotted for changes in DNAm and DNAhm only.

SUPPLEMENTARY FIGURE 10 Shared differential methylation and gene expression changes. **(A)** Venn diagram comparing genes annotated to DMCs (brainstem) or top ranked CpGs (hippocampus) and DEGs identified in brainstem or hippocampus across all contrast. Overlapping genes are shown. **(B)** Venn diagram comparing genes annotated to the union of top 1000 ranked CpGs across all contrasts and DEGs identified in brainstem and hippocampus. **(C)** Five most significant enriched Gene Ontology terms for overlapping genes identified for brainstem and hippocampus in **(B)** with indicated adjusted p-value, and gene count (BP: biological process). **(D)** Expression and methylation level for *Ndufs7* (cg28717035_BC21), *Igsf21* (cg41605845_BC21), and *Apoe* (cg44295218_BC21) as individual nRPKM data points and total methylation values across experimental groups with mean and standard error of the mean as horizontal and vertical line.

SUPPLEMENTARY FIGURE 11 SMITE identifies brain region-specific and cross-regional protein interaction networks. **(A)** Overview of all modules identified by SMITE analysis in brainstem and hippocampus. Each node represents a gene color-coded by its region-specific or cross-regional presence. **(B)** Protein interaction network for most significant module in hippocampus identified by SMITE analysis. Node color and edge thickness represents chi-square values derived from SMITE representing for the individual gene (node) or the gene connection (edge) how strongly they show evidence for coordinated expression and methylation changes. **(C)** Expression and methylation level for *Pcsk1n* (cg47430946_BC11 and cg47430915_TC21), *Ptprn* (cg37096612_BC21 and cg37096553_TC11), *Cdk5r2* (cg37092018_BC21), and *Cyb561* (cg30104400_BC21) as individual nRPKM data points and total methylation values across experimental groups with mean and standard error of the mean as horizontal and vertical line. **(D)** Venn diagram comparing number of genes identified in the semaphoring-plexin module in brainstem and hippocampus. **(E)** Circos plot of the spatial distribution of 31 shared genes found in the semaphoring-plexin module in brainstem and hippocampus.

SUPPLEMENTARY TABLE 1 Differentially methylated CpG sites in brainstem for each contrast.

SUPPLEMENTARY TABLE 2 Top 100 ranked CpG sites in hippocampus for each contrast.

SUPPLEMENTARY TABLE 3 Significant SMITE modules per brain region.
