# Supplementary material for: Loss of epigenetic adaptation to a high-fat diet in alpha-synuclein transgenic mice
Source: Front Cell Neurosci. 2026 Aug 5;20:1901103. doi: 10.3389/fncel.2026.1901103 (PMC13485732; doi:10.3389/fncel.2026.1901103)

Supplementary Figure 1

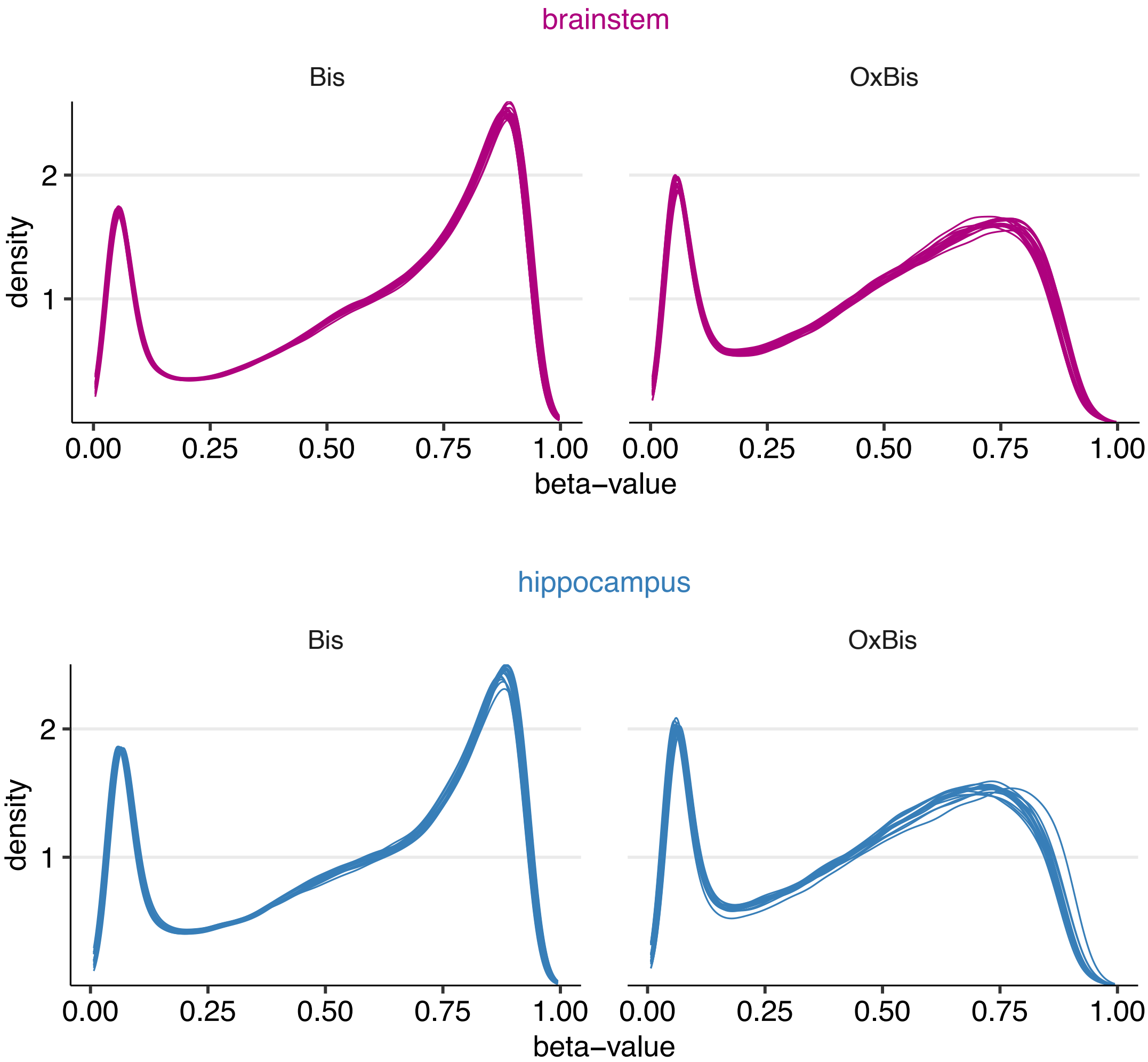

Supplementary Figure 2

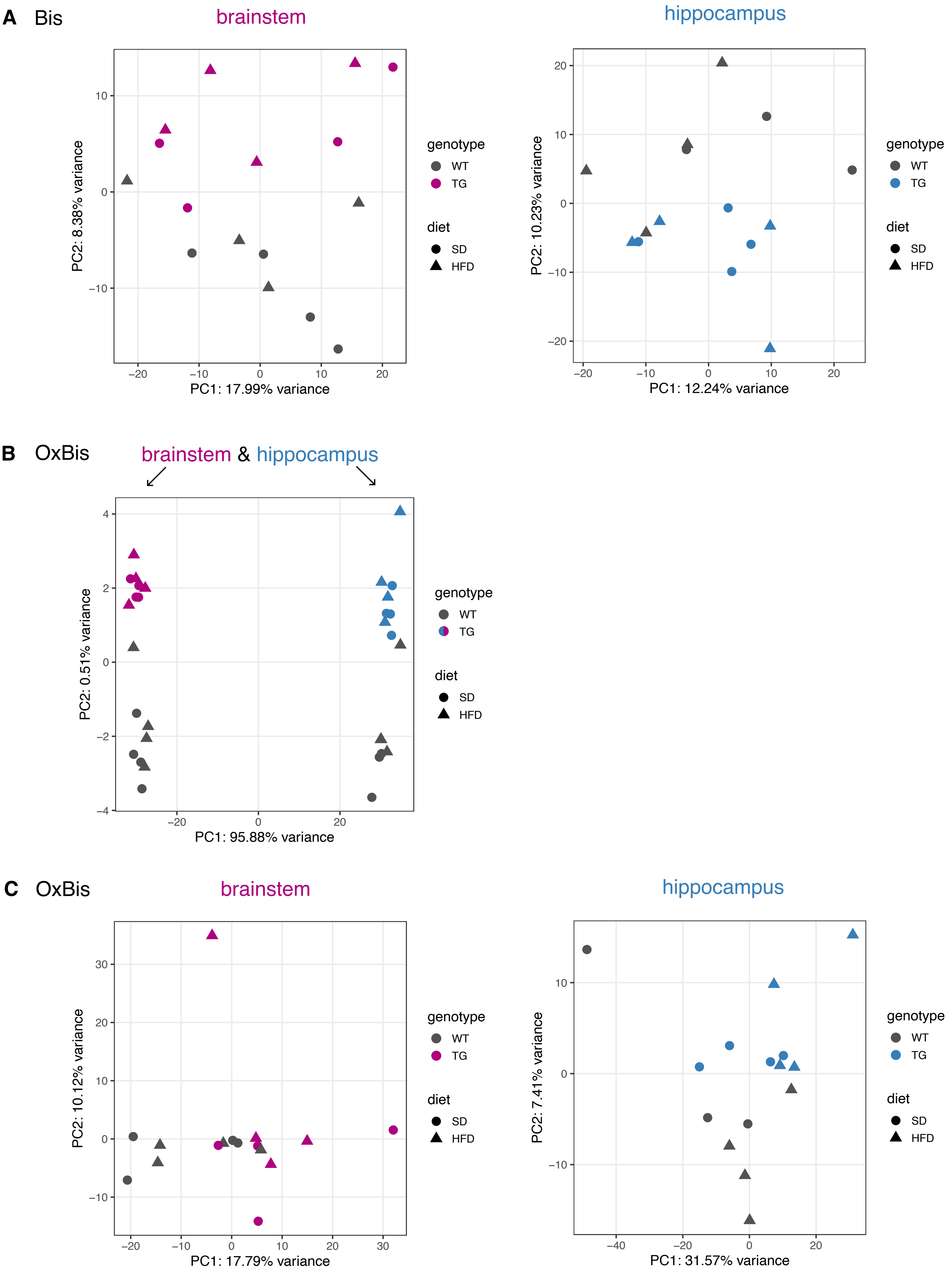

Supplementary Figure 3

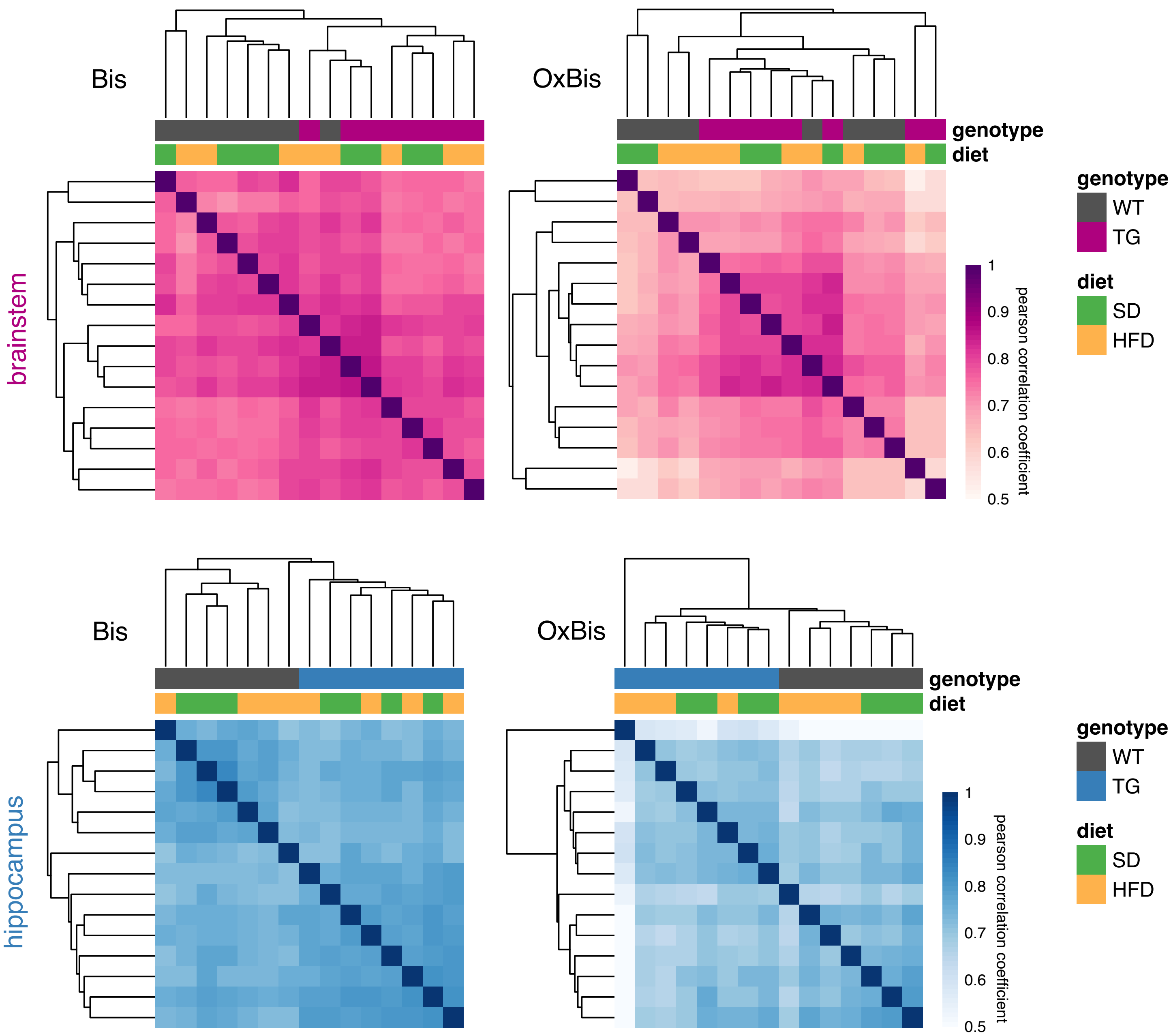

Supplementary Figure 4

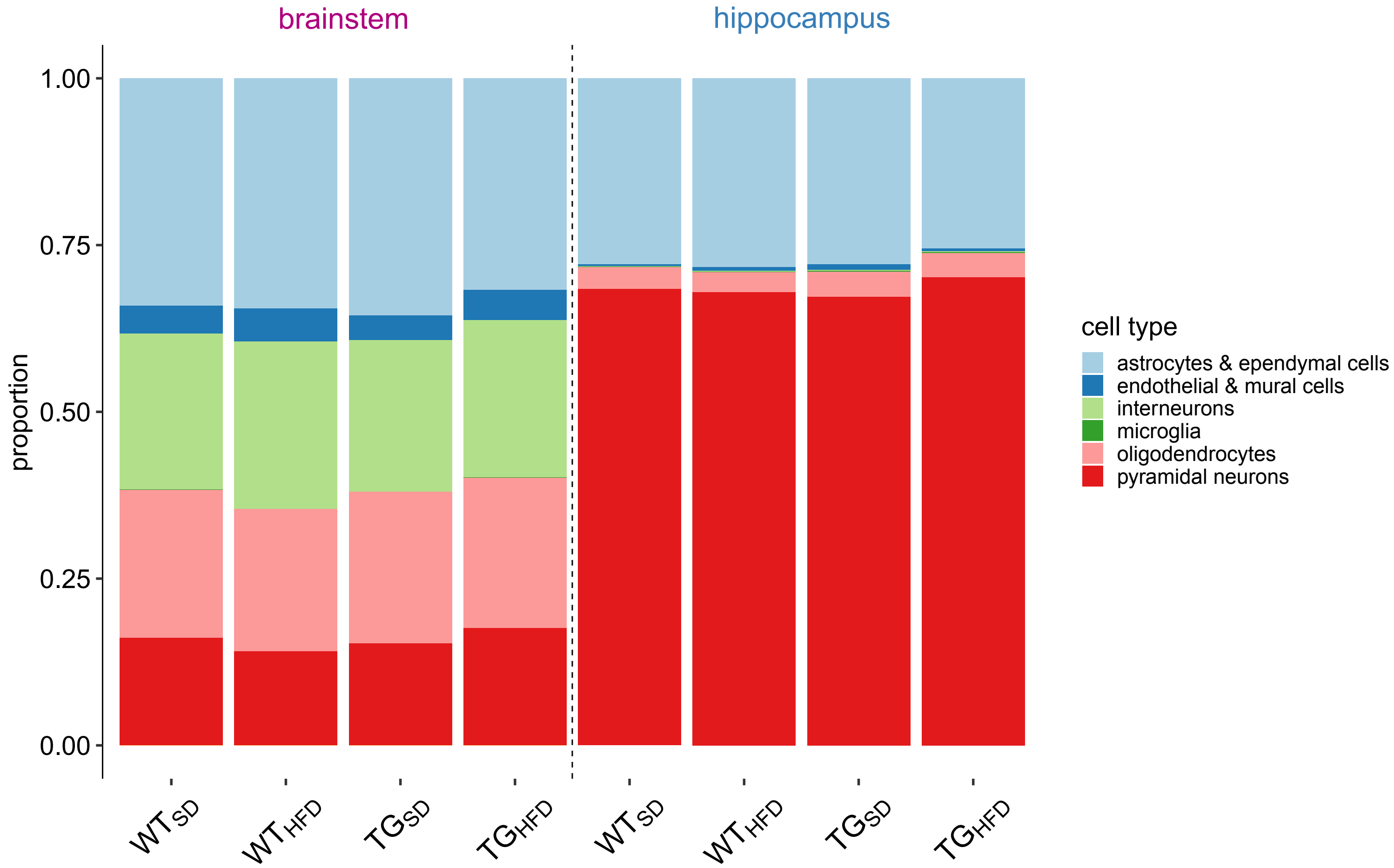

Supplementary Figure 5

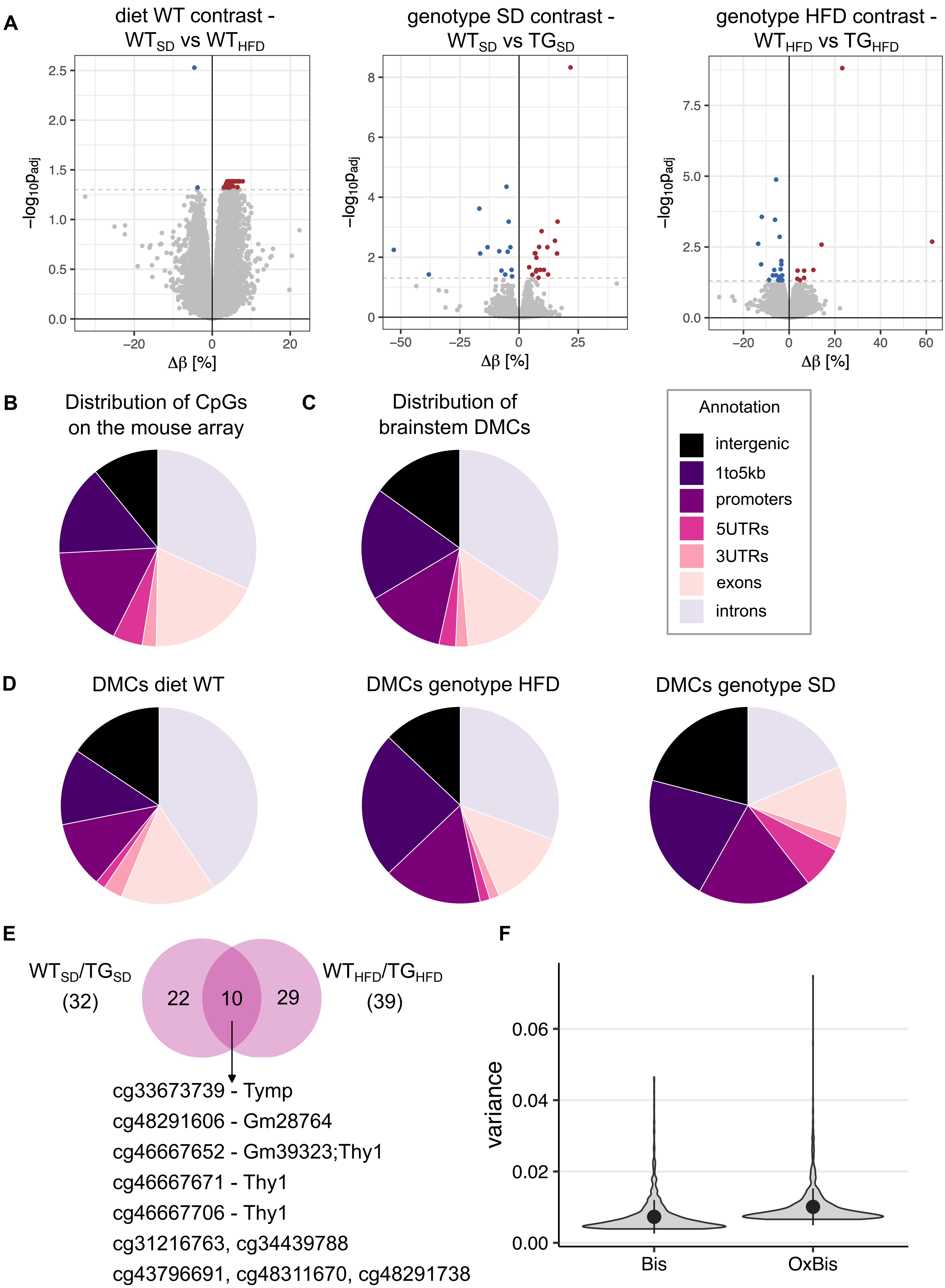

Supplementary Figure 6

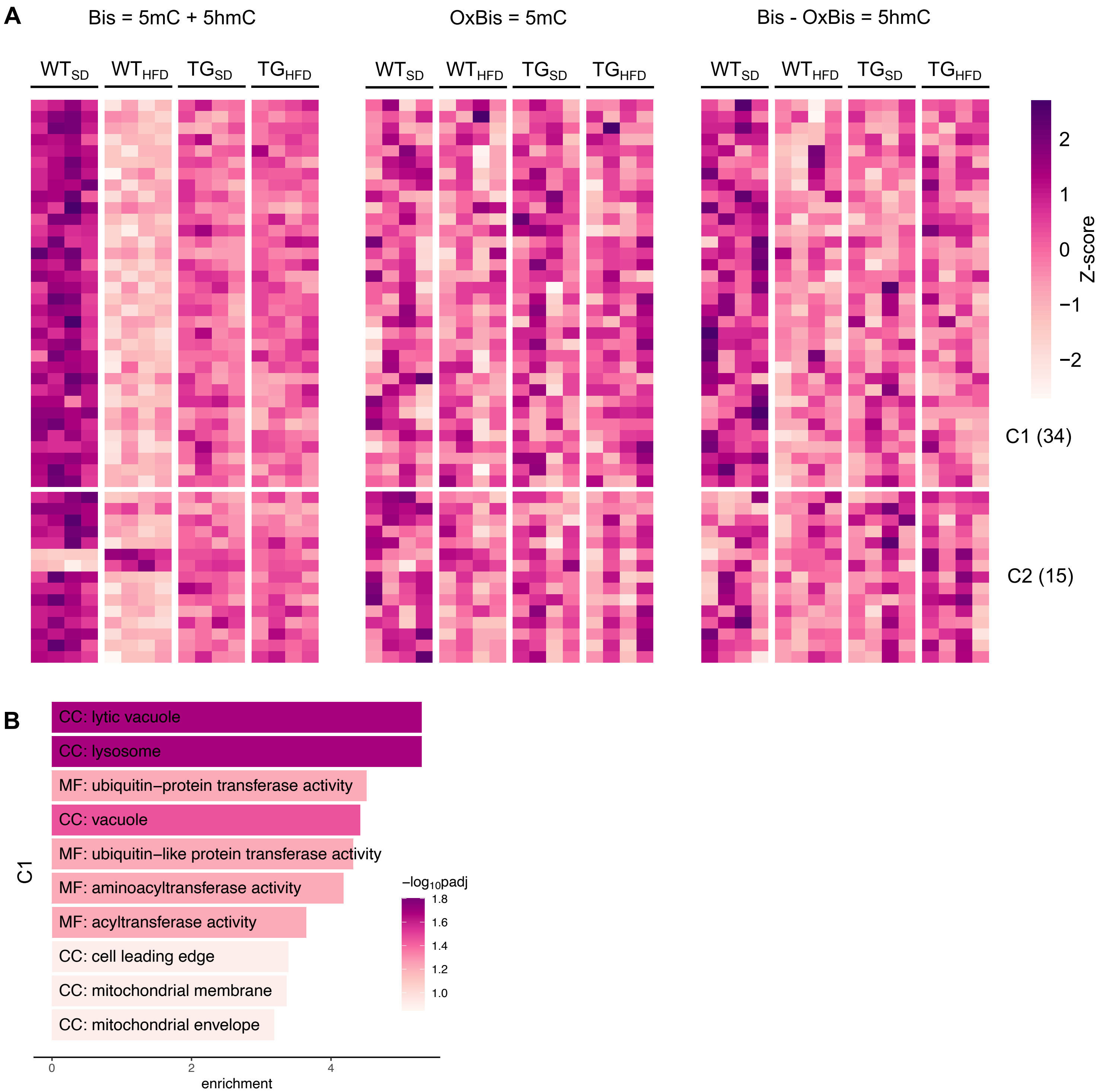

Supplementary Figure 7

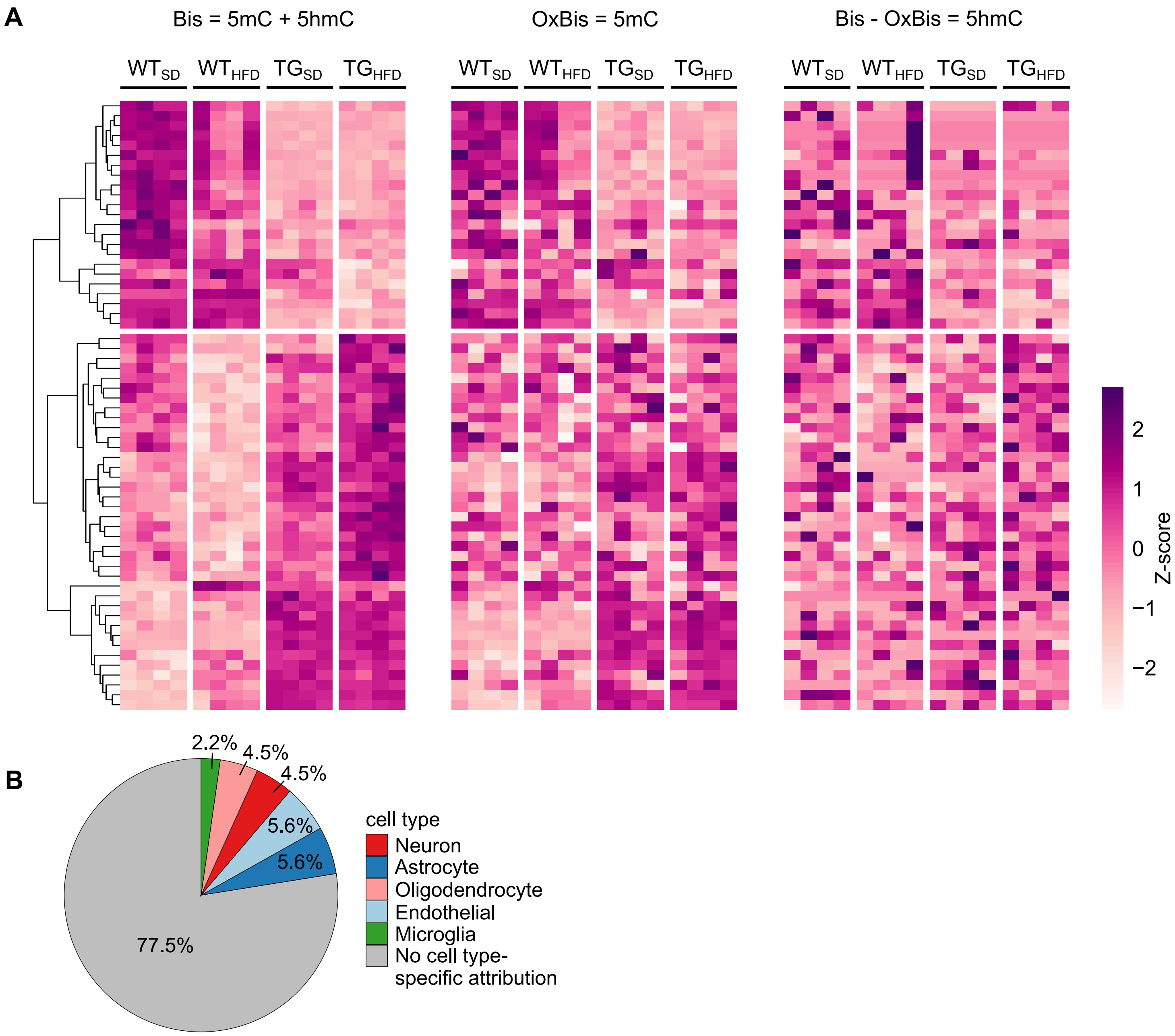

Supplementary Figure 8

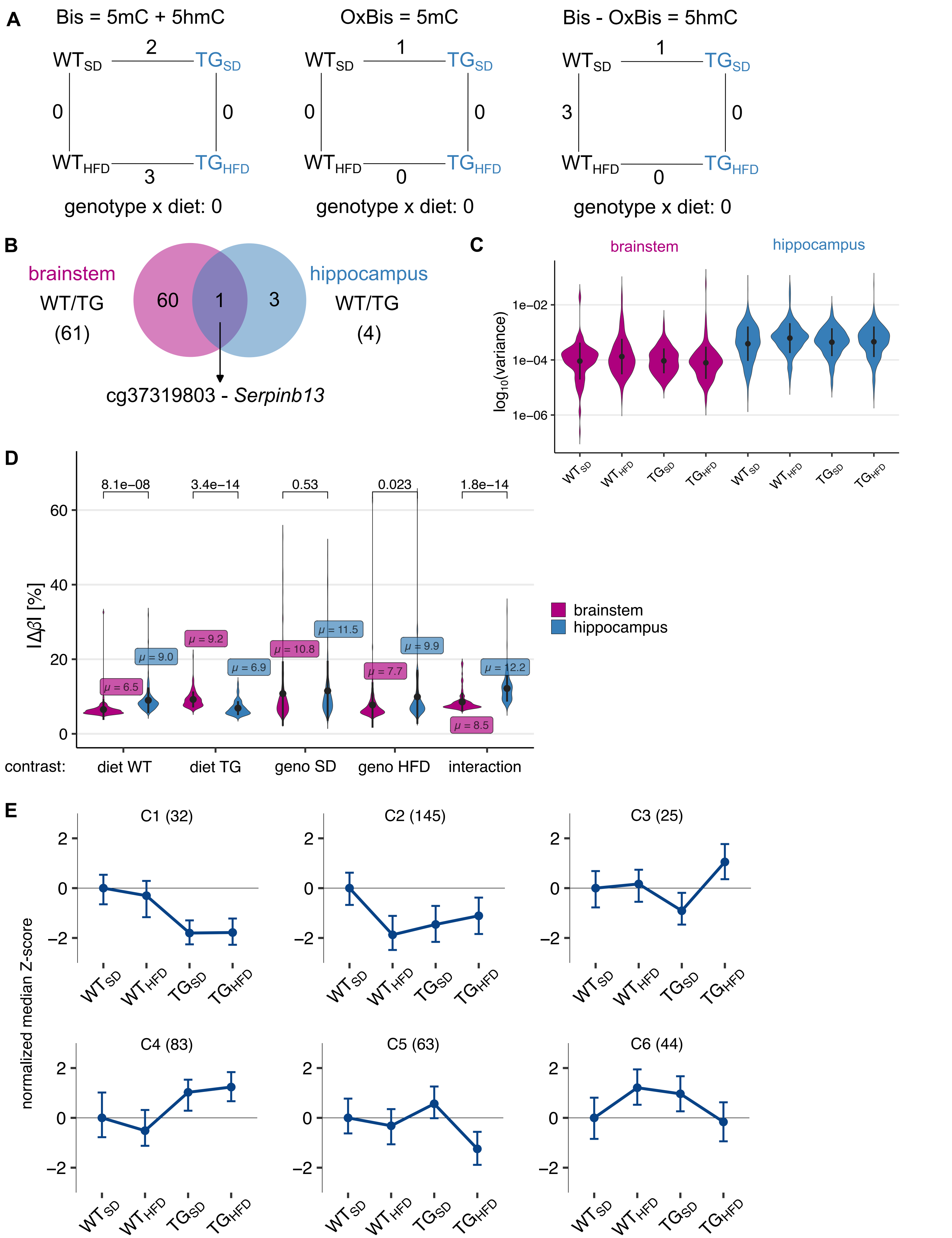

Supplementary Figure 9

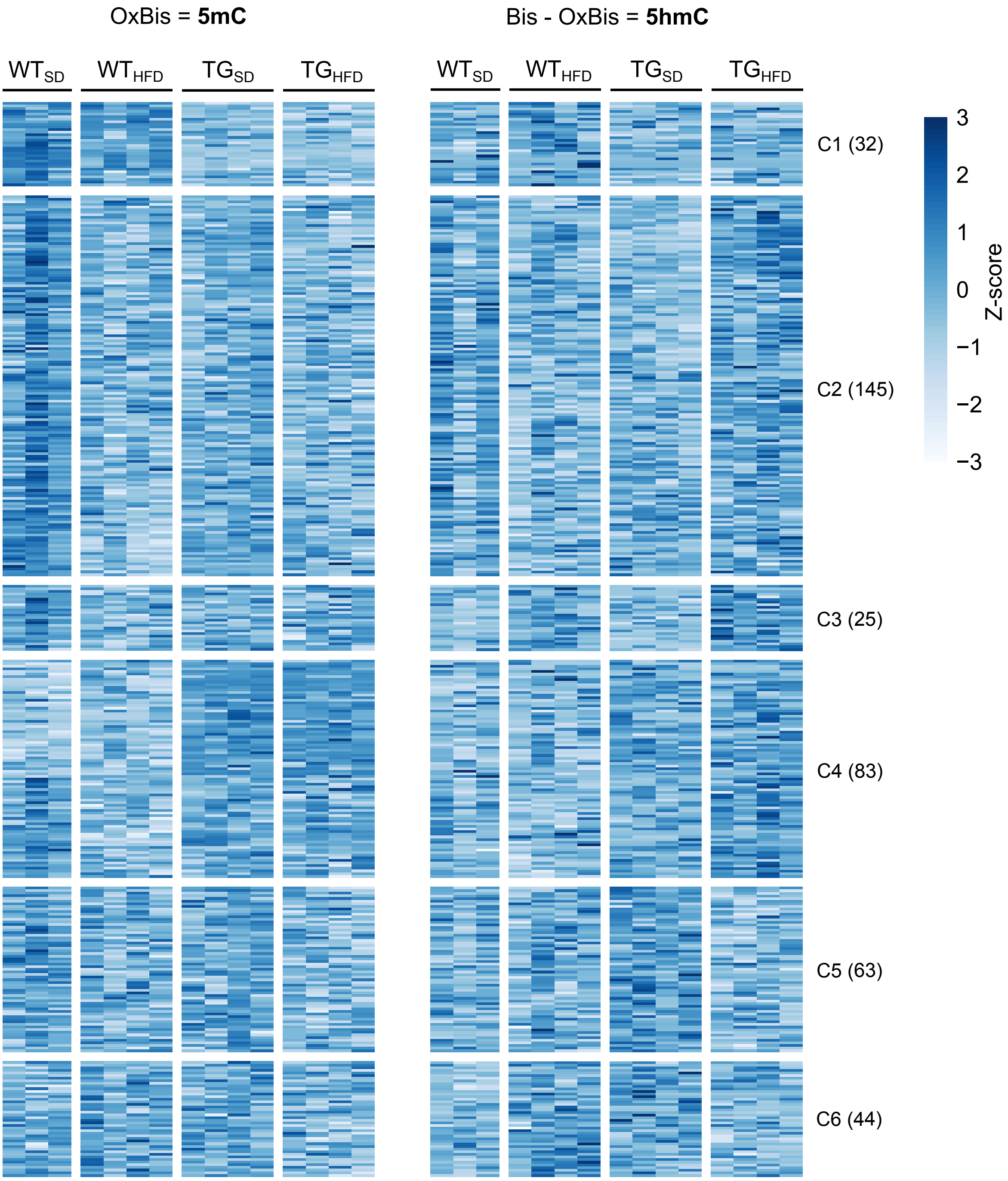

Supplementary Figure 10

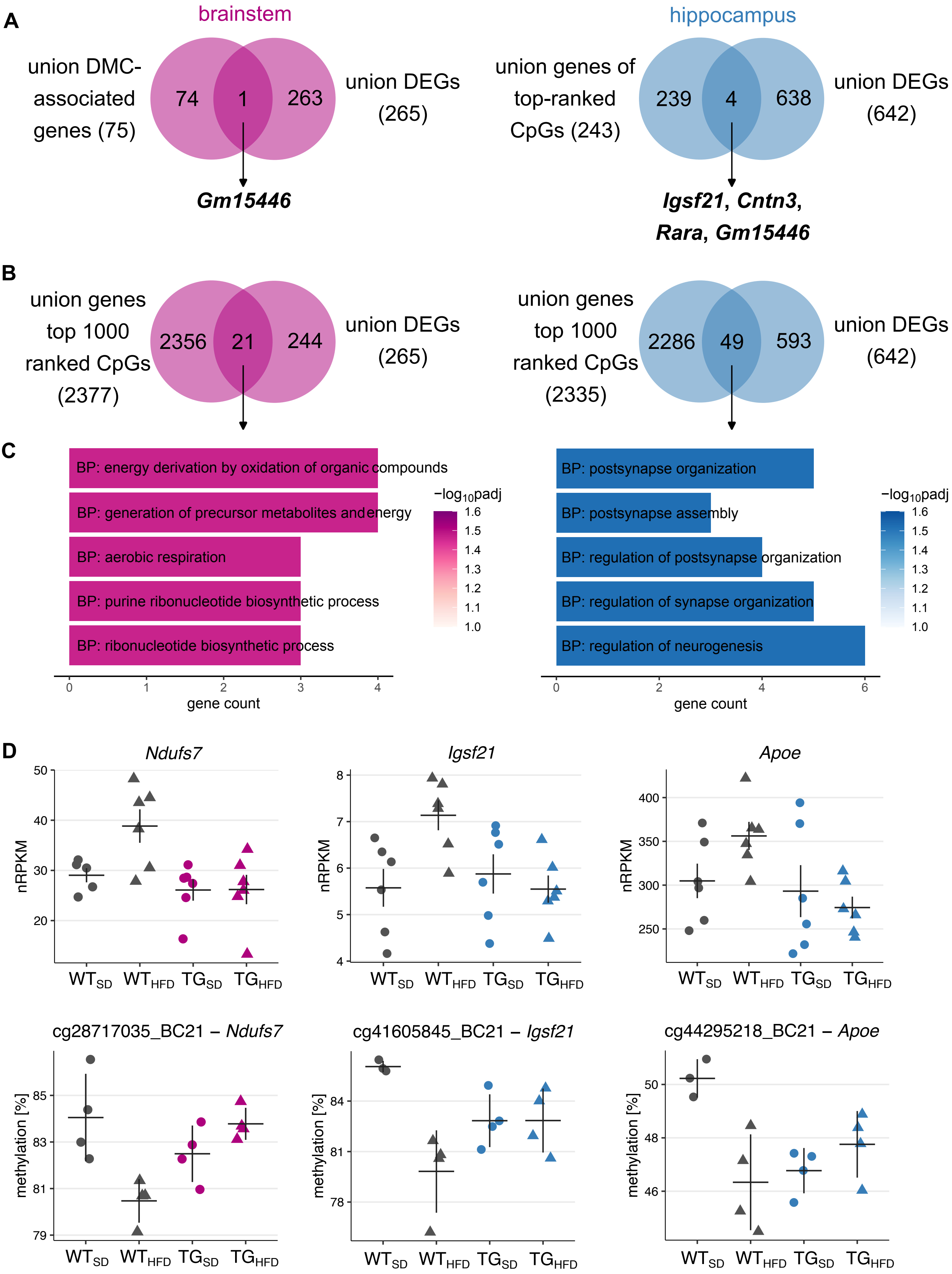

Supplementary Figure 11

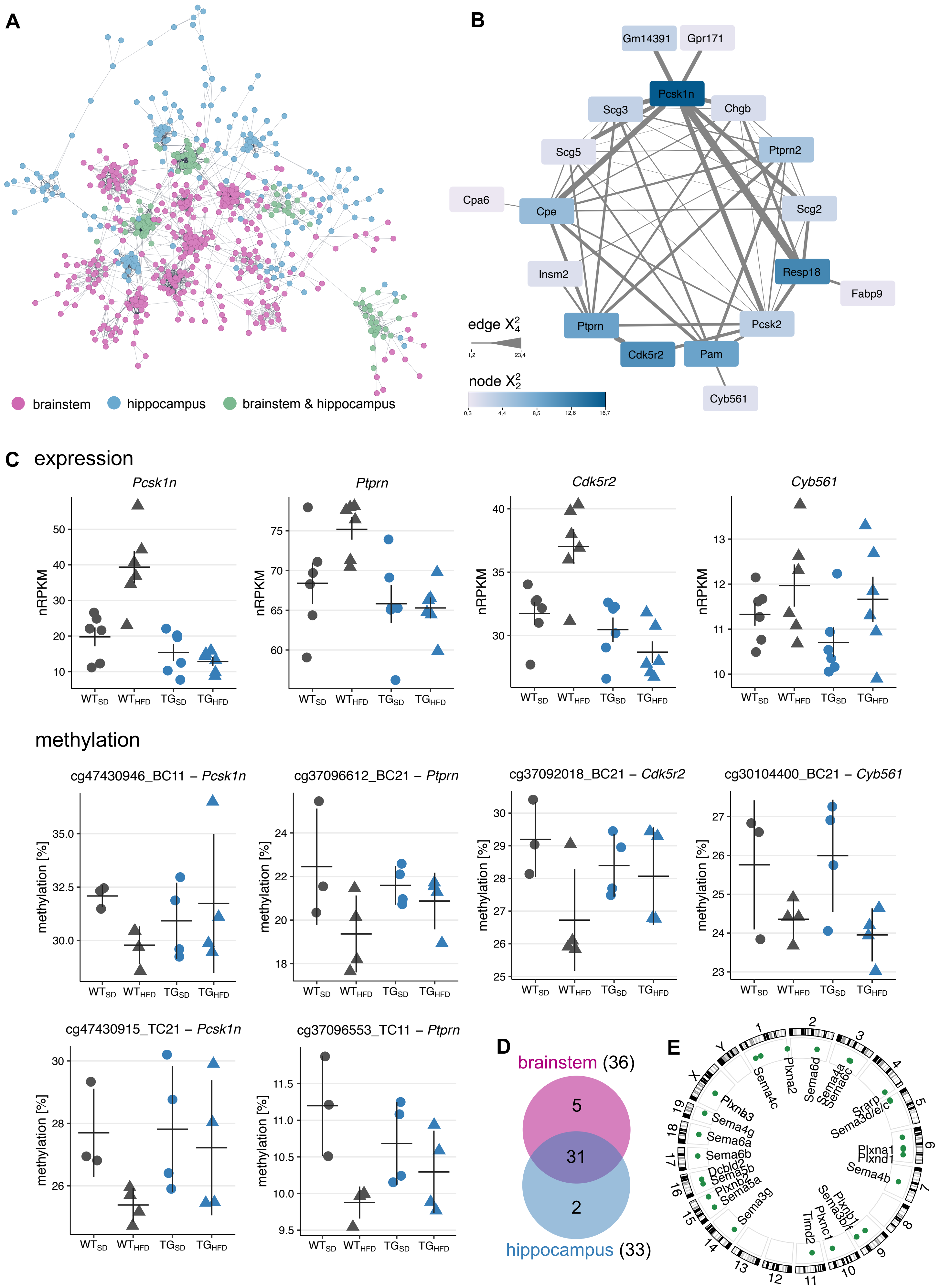

Supplement: Supplementary file 1 [file Data_Sheet_1.PDF]
